# Supplementary material for: Unique Huygens-Fresnel electromagnetic transportation of chiral Dirac wavelet in topological photonic crystal
Source: Nat Commun. 2023 Jun 2;14:3040. doi: 10.1038/s41467-023-38325-8 (PMC10238500; doi:10.1038/s41467-023-38325-8)
Supplement: Supplementary file 1 — Supplementary Information [file 41467_2023_38325_MOESM1_ESM.pdf]

Supplementary Information

Unique Huygens-Fresnel electromagnetic transportation of chiral Dirac wavelet in topological photonic crystal

Xing-Xiang Wang†, Zhiwei Guo†, Juan Song, Haitao Jiang, Hong Chen*, and Xiao Hu*

[1]International Center for Materials Nanoarchitectonics (WPI-MANA), National Institute for Materials Science; Tsukuba 305-0044, Japan.

[2]Graduate School of Science and Technology, University of Tsukuba; Tsukuba 305-8571, Japan.

[3]MOE Key Laboratory of Advanced Micro-Structured Materials, School of Physics Science and Engineering, Tongji University; Shanghai 200092, China.

*Corresponding authors' Emails: hongchen@tongji.edu.cn; Hu.Xiao@nims.go.jp

These authors contributed equally: Xing-Xiang Wang, Zhiwei Guo.

Contents:

Supplementary Note I. k · p Hamiltonian in LC circuits 2
Supplementary Note II. Interference between the p and d modes excited by a chiral wavelet 4
Supplementary Note III. Second-order perturbation for the $k \cdot p$ Hamiltonian in LC circuits 9
Supplementary Note IV. Configurations of the microstrip-based structures 14
Supplementary Note V. Experimental setup and details of measurement 14
Supplementary Note VI. Responses of microstrips to clockwise-phase-winding chiral source 15
Supplementary Note VII. Relation between Poynting vector and Berry phase 16
Supplementary Note VIII. Berry curvatures in lumped-element LC circuit 21
Supplementary Note IX. OAM of the excited modes 23
Supplementary Note X. Chiral response of the microstrip structures without cladding 24
Supplementary Note XI. Chiral response for source located at the C_{3v}-symmetric area 25
Supplementary Note XII. Negative refraction and left-handedness 26

Supplementary Note I. k · p Hamiltonian in LC circuits

The lumped-element LC circuit[1] for the honeycomb microstrip is schematically shown in Supplementary Fig. 1. The voltage on node i is given by

$$-\frac{1}{C}\sum_{j\in\{n.n.\}}\frac{1}{L_{ij}}\left(V_i-V_j\right)=\frac{\mathrm{d}^2V_i}{\mathrm{d}t^2}. \quad \text{(A1)}$$

Considering the harmonic modes with $\mathrm{d}^2V_i/\mathrm{d}t^2=-\omega^2V_i$, for the hexagonal unit cell shown in Supplementary Fig. 1c, one has the following secular equation

$$H\mathbf{V}=\omega^2\mathbf{V}, \quad \text{(A2)}$$

$$H=\omega_0^2\begin{bmatrix}2+\tau & -1 & 0 & -\tau X & 0 & -1\\ -1 & 2+\tau & -1 & 0 & -\tau Y & 0\\ 0 & -1 & 2+\tau & -1 & 0 & -\tau X^*Y\\ -\tau X^* & 0 & -1 & 2+\tau & -1 & 0\\ 0 & -\tau Y^* & 0 & -1 & 2+\tau & -1\\ -1 & 0 & -\tau XY^* & 0 & -1 & 2+\tau\end{bmatrix}, \quad \text{(A3)}$$

where ω is angular frequency, $\boldsymbol{V}=[V_1,V_2,V_3,V_4,V_5,V_6]^T$, $\omega_0^2=1/L_1C$, $\tau=L_1/L_2$, $X=\exp(i\mathbf{k}\cdot\mathbf{a}_1)$ and $Y=\exp(i\mathbf{k}\cdot\mathbf{a}_2)$. The eigen states of Hamiltonian (A3) at Γ point (See Supplementary Fig. 1d) are given by

$$|s\rangle=[1,\ 1,\ 1,\ 1,\ 1,\ 1]^T/\sqrt{6},$$

$$|p_x\rangle=[2,\ 1,\ -1,\ -2,\ -1,\ 1]^T/2\sqrt{3},$$

$$|p_y\rangle=[0,\ 1,\ 1,\ 0,\ -1,\ -1]^T/2,$$

$$|d_{x^2-y^2}\rangle=[2,\ -1,\ -1,\ 2,\ -1,\ -1]^T/2\sqrt{3},$$

$$|d_{2xy}\rangle=[0,\ 1,\ -1,\ 0,\ 1,\ -1]^T/2,$$

$$|f\rangle=[1,\ -1,\ 1,\ -1,\ 1,\ -1]^T/\sqrt{6}.$$

Expanding Hamiltonian (A3) to the lowest orders of $\mathbf{k}$, and projecting it to the subspace spanned by $(|p_+\rangle, |d_+\rangle, |p_-\rangle, |d_-\rangle)^T$, with $|p_\pm\rangle = |p_x\rangle \pm i|p_y\rangle$ and $|d_\pm\rangle = |d_{x^2-y^2}\rangle \pm i|d_{2xy}\rangle$, one has $H \to \omega_0^2(2+\tau)I_{4\times4} + \mathrm{Diag}(\widehat{H}_+, \widehat{H}_-)$, where $\widehat{H}_\pm$ is

$$\widehat{H}_\pm = \begin{bmatrix} -M - \frac{1}{4}\omega_0^2\tau a_0^2 k^2 & -\frac{i}{2}\omega_0^2\tau a_0 k_\pm \\ \frac{i}{2}\omega_0^2\tau a_0 k_\mp & M + \frac{1}{4}\omega_0^2\tau a_0^2 k^2 \end{bmatrix}, \tag{A4}$$

with $M = (1-\tau)\omega_0^2$. Note that M is the Dirac mass according to the convention of Dirac-type Hamiltonian which is independent of momentum. Here we only concentrate on the case of small Dirac mass around the transition point of topology $\tau = 1$, since otherwise higher orders of k are required in the following discussions.

By diagonalizing Hamiltonian (A4), one obtains the dispersion relation below the lower band edge around Γ point to the second order of k,

$$\omega_{\mathbf{k}}^2 = \omega_{\mathrm{edge}}^2 - \frac{\alpha}{2}\mathbf{k}^2, \tag{A5}$$

with

$$\omega_{\mathrm{edge}}^2 = (2 + \tau - |1-\tau|)\omega_0^2$$

and

$$\alpha = \frac{a_0^2\tau(2-\tau)}{4|1-\tau|}\omega_0^2.$$

Around Γ point, one has

$$\omega_{\mathbf{k}} = \sqrt{\omega_{\mathrm{edge}}^2 - \frac{\alpha}{2}\mathbf{k}^2} \approx \omega_{\mathrm{edge}} - \frac{\alpha}{4\omega_{\mathrm{edge}}}\mathbf{k}^2. \tag{A6}$$

The band mass m defined by

$$E_{\mathbf{k}} = \hbar\omega_{\mathbf{k}} = \hbar\omega_{\text{edge}} + \frac{\hbar^2\mathbf{k}^2}{2m}, \tag{A7}$$

is given by

$$m = -2\hbar\omega_{\text{edge}}/\alpha = -\frac{8\hbar|1-\tau|\sqrt{2+\tau-|1-\tau|}}{a_0^2\tau(2-\tau)\omega_0}.$$

The upwardly convex dispersion below the band gap with $\alpha > 0$ in Eq. (A5) specifies a negative band mass which yields negative refraction (*8*) in both topological ($\tau > 1$) and trivial ($\tau < 1$) photonic crystals (see Supplementary Note XII).

For $\tau \approx 1$ around the transition point of topology under concern, the band mass is given by

$$m \approx -\frac{8\sqrt{3}\hbar}{\omega_0 a_0^2}|1-\tau|. \tag{A8}$$

Therefore, although a transition in topology takes place when the Dirac mass $M = (1-\tau)\omega_0^2$ changes sign, the band mass remains unchanged as far as the lower band edge is concerned. When the Dirac mass goes to zero, the band mass approaches zero as well as it should since the dispersion relation becomes linear.

Supplementary Note II. Interference between the p and d modes excited by a chiral wavelet

We consider two neighbor unit cells in a shell at the radius $|\mathbf{R}| = R$ from the system center (see Supplementary Fig. 2), the wavefunction satisfies Bloch's theorem:

$$\psi_{\mathbf{k}}(\mathbf{R} + \mathbf{r} + \delta\mathbf{R}) = e^{i\mathbf{k}\cdot\delta\mathbf{R}}\psi_{\mathbf{k}}(\mathbf{R} + \mathbf{r}). \tag{A9}$$

According to rotational symmetry of the excited modes around the central unit cell:

$$\psi_{\mathbf{k}}(\mathbf{R}+\mathbf{r}+\delta\mathbf{R}) = \psi_{\mathbf{k}+\delta\mathbf{k}}(\mathbf{R}+\mathbf{r}), \tag{A10}$$

where $\delta\mathbf{k} = \delta\theta\mathbf{e}_z \times \mathbf{k}$. The R.H.S. of Eqs. (A9) and (A10) are equivalent:

$$e^{i\boldsymbol{k}\cdot\delta\boldsymbol{R}}\psi_{\boldsymbol{k}}(\boldsymbol{R}+\boldsymbol{r}) = \psi_{\boldsymbol{k}+\delta\boldsymbol{k}}(\boldsymbol{R}+\boldsymbol{r}). \tag{A11}$$

Expanding the R.H.S. of Eq. (A11) to the 1st order, one has:

$$e^{i\mathbf{k}\cdot\delta\mathbf{R}}\psi_{\mathbf{k}}(\mathbf{R}+\mathbf{r}) = \psi_{\mathbf{k}}(\mathbf{R}+\mathbf{r}) + \delta\mathbf{k}\cdot\nabla_{\mathbf{k}}\psi_{\mathbf{k}}(\mathbf{R}+\mathbf{r}), \tag{A12}$$

Because $\mathbf{k}$ is small for frequency close to the band edge locating at the Γ point, one has $e^{i\mathbf{k}\cdot\delta\mathbf{R}} \simeq 1 + i\mathbf{k}\cdot\delta\mathbf{R}$. Equation (A12) becomes

$$(1 + i\boldsymbol{k}\cdot\delta\boldsymbol{R})\psi_{\boldsymbol{k}}(\boldsymbol{R}+\boldsymbol{r}) = (1 + \delta\boldsymbol{k}\cdot\nabla_{\boldsymbol{k}})\psi_{\boldsymbol{k}}(\boldsymbol{R}+\boldsymbol{r}),$$

$$i\boldsymbol{k}\cdot\delta\boldsymbol{R}\psi_{\boldsymbol{k}}(\boldsymbol{R}+\boldsymbol{r}) = \delta\boldsymbol{k}\cdot\nabla_{\boldsymbol{k}}\psi_{\boldsymbol{k}}(\boldsymbol{R}+\boldsymbol{r}). \tag{A13}$$

For the L.H.S and R.H.S. of Eq. (A13) one has

$$\begin{aligned}\mathbf{k}\cdot\delta\mathbf{R} &= (k_R\mathbf{e}_R + k_\theta\mathbf{e}_\theta)\cdot(\delta\theta\mathbf{e}_z \times R\mathbf{e}_R)\\ &= (k_R\mathbf{e}_R + k_\theta\mathbf{e}_\theta)\cdot(R\delta\theta\mathbf{e}_\theta)\\ &= k_\theta R\delta\theta,\end{aligned}$$

and

$$\begin{aligned}\delta\mathbf{k}\cdot\nabla_{\mathbf{k}} &= [\delta\theta\mathbf{e}_z \times (k_R\mathbf{e}_R + k_\theta\mathbf{e}_\theta)]\cdot\left(\frac{\partial}{\partial k_R}\mathbf{e}_R + \frac{\partial}{\partial k_\theta}\mathbf{e}_\theta\right)\\ &= (-k_\theta\delta\theta\mathbf{e}_R + k_R\delta\theta\mathbf{e}_\theta)\cdot\left(\frac{\partial}{\partial k_R}\mathbf{e}_R + \frac{\partial}{\partial k_\theta}\mathbf{e}_\theta\right)\\ &= -k_\theta\delta\theta\frac{\partial}{\partial k_R} + k_R\delta\theta\frac{\partial}{\partial k_\theta},\end{aligned}$$

respectively.

The modes excited by a chiral source placed at the central unit cell are represented by eigenmodes corresponding to Hamiltonian (A4) near Γ point (first order perturbation with respect to k)

$$|P_+\rangle = |p_+\rangle - \frac{ivk_-}{2M}|d_+\rangle,$$

$$|D_+\rangle = |d_+\rangle - \frac{ivk_+}{2M}|p_+\rangle, \tag{A14}$$

where a global phase is absent, $M = 1 - \tau$, $v = \tau a_0/2$ and

$$\begin{aligned} k_\pm &= k_x \pm ik_y \\ &= |\mathbf{k}|e^{\pm i\theta_k} \\ &= |\mathbf{k}|e^{\pm i(\theta_k - \theta)}e^{\pm i\theta} \\ &= (k_R \pm ik_\theta)e^{\pm i\theta}. \end{aligned} \tag{A15}$$

Let $\psi_{\boldsymbol{k}}$ be $|P_+\rangle$ and multiply $\langle d_+|$ to the left of both sides of Eq. (A13) one has

$$\langle d_+|i\mathbf{k}\cdot\delta\mathbf{R}|P_+\rangle = \langle d_+|\delta\mathbf{k}\cdot\nabla_{\mathbf{k}}|P_+\rangle,$$

$$\langle d_+|ik_\theta R\delta\theta\left(|p_+\rangle - \frac{ivk_-}{2M}|d_+\rangle\right) = \langle d_+|\left(-k_\theta\delta\theta\frac{\partial}{\partial k_R} + k_R\delta\theta\frac{\partial}{\partial k_\theta}\right)\left(|p_+\rangle - \frac{ivk_-}{2M}|d_+\rangle\right),$$

$$ik_\theta R\delta\theta\left(-\frac{ivk_-}{2M}\right) = \left(-k_\theta\delta\theta\frac{\partial}{\partial k_R} + k_R\delta\theta\frac{\partial}{\partial k_\theta}\right)\left(-\frac{ivk_-}{2M}\right),$$

$$ik_\theta R\delta\theta k_- = \left(-k_\theta\delta\theta\frac{\partial}{\partial k_R} + k_R\delta\theta\frac{\partial}{\partial k_\theta}\right)k_-.$$

Inserting Eq. (A15) to the above equation one has

$$ik_\theta R\delta\theta k_- = \left(-k_\theta\delta\theta\frac{\partial}{\partial k_R} + k_R\delta\theta\frac{\partial}{\partial k_\theta}\right)(k_R - ik_\theta)e^{-i\theta},$$

$$ik_\theta R\delta\theta k_- = -k_\theta\delta\theta e^{-i\theta} + k_R\delta\theta\left(-ie^{-i\theta}\right),$$

$$ik_\theta R\delta\theta k_- = -i(k_R - ik_\theta)\delta\theta e^{-i\theta},$$

$$ik_\theta R\delta\theta k_- = -i\delta\theta k_-,$$

$$k_\theta R = -1. \tag{A16}$$

In the same way, let $\psi_{\boldsymbol{k}}$ be $|D_+\rangle$ and multiply $\langle p_+|$ to the left of both sides of Eq. (A13) one has

$$\langle p_+|i\mathbf{k}\cdot\delta\mathbf{R}|D_+\rangle = \langle p_+|\delta\mathbf{k}\cdot\nabla_\mathbf{k}|D_+\rangle,$$

$$\langle p_+|ik_\theta R\delta\theta\left(|d_+\rangle - \frac{ivk_+}{2M}|p_+\rangle\right) = \langle p_+|\left(-k_\theta\delta\theta\frac{\partial}{\partial k_R} + k_R\delta\theta\frac{\partial}{\partial k_\theta}\right)\left(|d_+\rangle - \frac{ivk_+}{2M}|p_+\rangle\right).$$

Following the same procedure as given in Eq. (A16), one has

$$k_\theta R = 1. \tag{A17}$$

Equations (A16) and (A17) indicate that for mode $|P_+\rangle$ one has $k_\theta < 0$ while for $|D_+\rangle$ one has $k_\theta > 0$.

For the purpose to investigate the response in tangential direction, it is natural to consider the case with zero radial component k_R, where one has $k_\theta = -|k|$ for $|P_+\rangle$ and $k_\theta = |k|$ for $|D_+\rangle$. According to Eq. (A13), one has

$$|P_+\rangle = |p_+\rangle - \frac{ivk_-}{2M}|d_+\rangle,$$

$$= |p_+\rangle + \frac{v|k|}{2M}e^{-i\theta}|d_+\rangle$$

$$|D_+\rangle = |d_+\rangle - \frac{ivk_+}{2M}|p_+\rangle,$$

$$= |d_+\rangle + \frac{v|k|}{2M}e^{i\theta}|p_+\rangle. \tag{A18}$$

In the trivial system where $M > 0$, $|p_+\rangle = \exp(i\phi)$ and $|d_+\rangle = \exp(2i\phi)$ enhance (suppress) each other at $\phi = \theta$ ($\phi = \theta + \pi$), *i.e.* the outer (inner) side of the unit cell seen from the system center, which indicates that globally a net EM transportation circulates counterclockwise around

the system center, in the same direction of the chiral source. In the topological system where $M < 0$, $|p_+\rangle$ and $|d_+\rangle$ enhance (suppress) each other at the inner (outer) side of individual unit cells, which results in a net EM transportation circulating clockwise around to the system center, in the opposite direction of the chiral source. This can be seen more explicitly by looking at the Poynting vectors.

The time-averaged local Poynting vector for a TM mode is given by

$$\begin{aligned}\langle \mathbf{S} \rangle &= \frac{1}{2\mu_0\omega}\mathrm{Im}\{E_z^*\nabla E_z\} \\ &= \frac{1}{2\mu_0\omega}\mathrm{Im}\left\{\mathbf{e}_r E_z^* \frac{\partial}{\partial r}E_z + \mathbf{e}_\phi \frac{1}{r}\, E_z^* \frac{\partial}{\partial \phi}E_z\right\} \end{aligned} \tag{A19}$$

For state $|P_+\rangle = |p_+\rangle - (ivk_+/2M)|d_+\rangle$ with $|p_+\rangle = |E_z|e^{i\phi}$ and $|d_+\rangle = |E_z|e^{2i\phi}$, the tangential component of Poynting vector at a point $\mathbf{r} = (r, \phi)$ inside the unit cell with center at $\mathbf{R} = (R, \theta)$ is evaluated as

$$\begin{aligned}\langle S_\phi \rangle &= \frac{1}{2\mu_0\omega r}\mathrm{Im}\left\{E_z^* \frac{\partial}{\partial \phi}E_z\right\} \\ &= \frac{|E_z|^2}{2\mu_0\omega r}\mathrm{Im}\left\{\left[e^{-i\phi} + \frac{ivk_+}{2M}e^{-2i\phi}\right]\frac{\partial}{\partial \phi}\left[e^{i\phi} - \frac{ivk_-}{2M}e^{2i\phi}\right]\right\} \\ &= \frac{|E_z|^2}{2\mu_0\omega r}\mathrm{Im}\left\{\left[e^{-i\phi} + \frac{ivk_+}{2M}e^{-2i\phi}\right]\left[ie^{i\phi} + \frac{vk_-}{M}e^{2i\phi}\right]\right\} \\ &= \frac{|E_z|^2}{2\mu_0\omega r}\mathrm{Im}\left\{i + i\cdot 2\left(\frac{v|k|}{2M}\right)^2 - \frac{vk_+}{2M}e^{-i\phi} + \frac{vk_-}{M}e^{i\phi}\right\} \\ &= \frac{|E_z|^2}{2\mu_0\omega r}\mathrm{Im}\left\{i + i\cdot 2\left(\frac{vk}{2M}\right)^2 - \frac{v(k_R + ik_\theta)}{2M}e^{-i(\phi-\theta)} + \frac{v(k_R - ik_\theta)}{M}e^{i(\phi-\theta)}\right\} \\ &= \frac{|E_z|^2}{2\mu_0\omega r}\left\{1 + 2\left(\frac{vk}{2M}\right)^2 - \frac{3v}{2M}[k_\theta\cos(\phi-\theta) - k_R\sin(\phi-\theta)]\right\}. \end{aligned} \tag{A20}$$

Similarly, for state $|D_+\rangle = |d_+\rangle - (ivk_+/2M)|p_+\rangle$ one has

$$\langle S_\phi \rangle = \frac{|E_z|^2}{2\mu_0 \omega r}\left\{2 + \left(\frac{v|k|}{2M}\right)^2 + \frac{3v}{2M}[k_\theta \cos(\phi-\theta) - k_R \sin(\phi-\theta)]\right\}. \quad \text{(A21)}$$

When $k_R = 0$, one has

$$\langle S_\phi \rangle = \frac{|E_z|^2}{2\mu_0 \omega r}\left\{1 + 2\left(\frac{v|k|}{2M}\right)^2 + \frac{3v|k|}{2M}\cos(\phi-\theta)\right\} \quad \text{(A22)}$$

for state $|P_+\rangle$, and

$$\langle S_\phi \rangle = \frac{|E_z|^2}{2\mu_0 \omega r}\left\{2 + \left(\frac{v|k|}{2M}\right)^2 + \frac{3v|k|}{2M}\cos(\phi-\theta)\right\} \quad \text{(A23)}$$

for state $|D_+\rangle$.

Supplementary Note III. Second-order perturbation for the $\boldsymbol{k}\cdot\boldsymbol{p}$ Hamiltonian in LC circuits

Expanding Hamiltonian (A3) to the second order of $\boldsymbol{k}$, and projecting it to the subspace spanned by the four states around the band gap $(|p_+\rangle, |d_+\rangle, |p_-\rangle, |d_-\rangle)^T$, with $|p_\pm\rangle = |p_x\rangle \pm i|p_y\rangle$ and $|d_\pm\rangle = |d_{x^2-y^2}\rangle \pm i|d_{2xy}\rangle$, one has $H \to \omega_0^2(2+\tau)I_{4\times4} + \widehat{H}$ with

$$\widehat{H} = \begin{bmatrix} -M-uk^2 & -ivk_+ & -\frac{1}{2}uk_-^2 & 0 \\ ivk_- & M+uk^2 & 0 & \frac{1}{2}uk_+^2 \\ -\frac{1}{2}uk_+^2 & 0 & -M-uk^2 & -ivk_- \\ 0 & \frac{1}{2}uk_-^2 & ivk_+ & M+uk^2 \end{bmatrix}, \quad \text{(A24)}$$

where $M = (1-\tau)\omega_0^2$, $u = \omega_0^2 a_0^2 \tau/4$, $v = \omega_0^2 a_0 \tau/2$, $k_\pm = k_x \pm i k_y = ke^{\pm i\theta}$ and $k^2 = k_+ k_-$. It is noticed that the terms $uk_\pm^2$ couple the two pseudospin sectors as can be seen clearly by comparing Eq. (1) in the main text and Eq. (A24), and that the coupling between $|d_+\rangle$ and $|d_-\rangle$ is in the order of k^2 due to the $C_{6\mathrm{v}}$ symmetry.

In order to perform the second-order perturbation analysis for Hamiltonian (A24) with k being small where the unperturbed Hamiltonian for $k = 0$ is doubly degenerate, we apply a unitary transformation from the basis $(|p_+\rangle, |d_+\rangle, |p_-\rangle, |d_-\rangle)^T$ to $(|p_1\rangle, |p_2\rangle, |d_1\rangle, |d_2\rangle)^T$ with

$$|p_1\rangle = |p_+\rangle + e^{2i\theta}|p_-\rangle, \tag{A25}$$

$$|p_2\rangle = |p_+\rangle - e^{2i\theta}|p_-\rangle, \tag{A26}$$

$$|d_1\rangle = |d_+\rangle + e^{-2i\theta}|d_-\rangle, \tag{A27}$$

$$|d_2\rangle = |d_+\rangle - e^{-2i\theta}|d_-\rangle, \tag{A28}$$

where $k_\pm = ke^{\pm i\theta}$. Under the new basis $(|p_1\rangle, |p_2\rangle, |d_1\rangle, |d_2\rangle)^T$ Hamiltonian $\widehat{H}$ in Eq. (A24) becomes

$$\widehat{H} \to \widetilde{H} = H_0 + H', \tag{A29}$$

with the unperturbed Hamiltonian

$$H_0 = \mathrm{Diag}(-M, -M, M, M), \tag{A30}$$

and the perturbation

$$H' = \begin{bmatrix} -\frac{3}{2}uk^2 & 0 & -ivke^{-2i\theta}\cos 3\theta & vke^{-2i\theta}\sin 3\theta \\ 0 & -\frac{1}{2}uk^2 & vke^{-2i\theta}\sin 3\theta & -ivke^{-2i\theta}\cos 3\theta \\ ivke^{2i\theta}\cos 3\theta & vke^{2i\theta}\sin 3\theta & \frac{3}{2}uk^2 & 0 \\ vke^{2i\theta}\sin 3\theta & ivke^{2i\theta}\cos 3\theta & 0 & \frac{1}{2}uk^2 \end{bmatrix}, \tag{A31}$$

where the double degeneracy has been removed. For the unperturbed eigenstate $|p_1\rangle$ in Eq. (A25), the eigenvalue and eigenwave function up to the second-order perturbation are given by

$$E_{P_1} = E_{p_1} + \langle p_1|H'|p_1\rangle + \frac{|\langle d_1|H'|p_1\rangle|^2}{E_{p_1} - E_{d_1}} + \frac{|\langle d_2|H'|p_1\rangle|^2}{E_{p_1} - E_{d_2}}$$

$$= -M - \left(\frac{3u}{2} + \frac{v^2}{2M}\right)k^2 + O(k^4), \tag{A32}$$

and

$$|P_1\rangle = |p_1\rangle + \sum_{l=1,2} |d_l\rangle \frac{\langle d_l|H'|p_1\rangle}{E_{p_1} - E_{d_l}} + \sum_{l=1,2}\sum_{m=1,2} |d_l\rangle \frac{\langle d_l|H'|d_m\rangle\langle d_m|H'|p_1\rangle}{\left(E_{p_1} - E_{d_l}\right)\left(E_{p_1} - E_{d_m}\right)}$$

$$+ \sum_{l=1,2} |d_l\rangle \frac{\langle d_l|H'|p_1\rangle\langle p_1|H'|p_1\rangle}{\left(E_{p_1} - E_{d_l}\right)^2}$$

$$= |p_+\rangle + e^{2i\theta}|p_-\rangle - \frac{ivk_-}{2M}|d_+\rangle - \frac{ivk_+}{2M}e^{2i\theta}|d_-\rangle + O(k^3). \tag{A33}$$

In the same way, for the unperturbed eigenstate $|p_2\rangle$ in Eq. (A26) one has

$$E_{P_2} = -M - \left(\frac{u}{2} + \frac{v^2}{2M}\right)k^2 + O(k^4), \tag{A34}$$

and

$$|P_2\rangle = |p_+\rangle - e^{2i\theta}|p_-\rangle - \frac{ivk_-}{2M}|d_+\rangle + \frac{ivk_+}{2M}e^{2i\theta}|d_-\rangle + O(k^3). \tag{A35}$$

It is important to see that, in Eqs. (A33) and (A35), the first and third terms form the pseudospin-up state, whereas the second and fourth terms form the pseudospin-down state given by Eq. (3) in the main text of manuscript derived by the first-order perturbation. The couplings between the two pseudospin sectors mix the two pseudospin states with equal weight in the form of bonding and antibonding pair with a gauge factor $e^{2i\theta}$, upon which the degeneracy of the two pseudospin states

is lifted as seen in Eqs. (A32) and (A34). Around Γ point the eigenvalues in Eqs. (A32) and (A34) match well with the frequency diagrams shown in Fig. 3(e,f,g) in the main text of manuscript derived numerically from the LC circuit model. It is also worth noticing that the way how the pseudospin-up and -down states evolve into two non-degenerate dispersions addressed above in the vicinity around Γ point coincides with the discussions in Ref. 2.

When a source with $+2\pi$ phase winding and a given frequency is introduced into a single hexagonal unite cell in the real-space system, the two eigenstates $|P_1(k)\rangle$ and $|P_2(k)\rangle$ with the same phase for $|p_+\rangle$ as appearing in Eqs. (A33) and (A35) should be excited simultaneously in the form of equal weight,

$$\frac{|P_1(k')\rangle + |P_2(k'')\rangle}{2} = |p_+\rangle - \frac{k' + k''}{2} \cdot \frac{ive^{-i\theta}}{2M}|d_+\rangle - \frac{k' - k''}{2} \cdot \frac{ive^{3i\theta}}{2M}|d_-\rangle + O(k^3), \quad \text{(A36)}$$

where the momenta k' and k'' are slightly different from each other due to the frequency splitting in Eqs. (A32) and (A34). It is intriguing to notice that in this process $|p_-\rangle$ vanishes completely due to cancellation. From Eqs. (A32) and (A34), it is straightforward to see that

$$\frac{k' - k''}{k' + k''} \approx \frac{1 - \tau}{2} \quad \text{(A37)}$$

is small around the transition point of $p - d$ band inversion under concern, so that the third term in Eq. (A36) can be dropped safely. We thus arrive at

$$\frac{|P_1(k')\rangle + |P_2(k'')\rangle}{2} \approx |p_+\rangle - \frac{iv\bar{k}_-}{2M}|d_+\rangle, \quad \text{(A38)}$$

with $\bar{k} \equiv (k' + k'')/2$, which takes the same form as $|P_+\rangle$ in Eq. (3) in the main text of our manuscript. Namely, the chiral source of $+2\pi$ phase winding excites the spinor wavefunction $|P_+\rangle$ in the pseudospin-up subspace obtained in the first-order perturbation.

When the system is excited by a source with -2π phase winding located in a single hexagonal unite cell, the two eigenstates $|P_1(k)\rangle$ and $|P_2(k)\rangle$ in Eqs. (A33) and (A35) should adapt to the source by changing their gauges such that $|p_-\rangle$ becomes synchronized with the source. It is straightforwardly to see that in this case $|p_+\rangle$ is suppressed totally, and $|d_+\rangle$ acquires a small coefficient and can be dropped, which yields a relation similar to Eq. (A33) for down pseudospin.

Following the same procedures, one can obtain the perturbed eigenvalues and wavefunctions for $|d_1\rangle$ and $|d_2\rangle$ in Eqs. (A26) and (A27)

$$E_{D_1} = M + \left(\frac{3u}{2} + \frac{v^2}{2M}\right)k^2 + O(k^4), \tag{A39}$$

$$|D_1\rangle = |d_+\rangle + e^{-2i\theta}|d_-\rangle - \frac{ivk_+}{2M}|p_+\rangle - \frac{ivk_-}{2M}e^{-2i\theta}|p_-\rangle + O(k^3), \tag{A40}$$

$$E_{D_2} = M + \left(\frac{u}{2} + \frac{v^2}{2M}\right)k^2 + O(k^4), \tag{A41}$$

$$|D_2\rangle = |d_+\rangle - e^{-2i\theta}|d_-\rangle - \frac{ivk_+}{2M}|p_+\rangle + \frac{ivk_-}{2M}e^{-2i\theta}|p_-\rangle + O(k^3). \tag{A42}$$

When a single-frequency source with $+4\pi$ phase winding is introduced at a single hexagonal unit cell, $|D_1(k')\rangle$ and $|D_2(k'')\rangle$ with the same phase of $|d_+\rangle$ are excited coherently,

$$\begin{aligned}\frac{|D_1(k_1)\rangle + |D_2(k_2)\rangle}{2} &= |d_+\rangle - \frac{k' + k''}{2}\cdot\frac{ive^{i\theta}}{2M}|p_+\rangle - \frac{k' - k''}{2}\cdot\frac{ive^{-3i\theta}}{2M}|p_-\rangle + O(k^3)\\ &\approx |d_+\rangle - \frac{iv\bar{k}_+}{2M}|p_+\rangle.\end{aligned} \tag{A43}$$

This takes the same form as $|D_+\rangle$ in Eq. (3) in the main text of our manuscript. Namely, the chiral source of $+4\pi$ phase winding excites the spinor wavefunction $|D_+\rangle$ in the pseudospin-up subspace

obtained in the first-order perturbation. The response to the source with -4π phase winding is obtained by the same consideration for the source of -2π phase winding.

This ensures that the theoretical discussions on responses to the chiral source based on the first-order perturbation are valid as far as frequency is close to the band edges.

Supplementary Note IV. Configurations of the microstrip-based structures

The structures with $M > 0$ and $M < 0$ are designed by loading lumped elements into the 2D honeycomb microstrip systems, as shown in Supplementary Fig. 3. The zoomed-in unit cells of the structures with $M > 0$ and $M < 0$ are displayed in the insets of Supplementary Fig. 3a and Supplementary Fig. 3b, respectively. Note $w_1 > w_2$ for the structure with $M > 0$, whereas $w_1 < w_2$ for the structure with $M < 0$[1,3].

Supplementary Note V. Experimental setup and details of measurement

The experimental setup is composed of a vector network analyzer, a 2D translational stage, a power divider, delay lines and the sample to be measured, which is shown in Supplementary Fig. 4. The signals are generated from the port 1 of a vector network analyzer (Agilent PNA Network Analyzer N5222A), and another antenna (i.e., near-filed probe) connecting to the port 2 of analyzer are employed to measure the electric fields. In order to construct the chiral source with special phase distribution, the signal generated from the port 1 is divided into six channels through a 1-6 microwave power divider, and then two external integrated circuits and extra transmission line systems are exploited to modulate the phase delays of six lumped input ports[1,4]. Especially, the six-antenna arrays with the phase of electromagnetic wave changing by 60 degrees and 120 degrees

between neighbor antennas induced by delayed lines are adopted respectively for the 2π- and 4π-phase winding chiral sources, as shown in Supplementary Fig. 5a and Supplementary Fig. 5b, respectively. The scale of voltage port arrays is still in the subwavelength scale near the operation frequency 1.42 GHz.

The samples are all fabricated on copper-clad 1.6 mm thick F4B substrates using laser direct structuring technology (LPKF ProtoLaser 200). In the experiment, the sample is put on a 10-cm-thick foam substrate with a permittivity of near one and then placed on an automatic translation device with scanning steps of 1 mm, which makes it feasible to accurately probe the field distribution using a near-field scanning measurement. An electric probe (shielded-rod antenna) of 5 mm length connecting to the port 2 of analyzer is vertically placed 1mm above the samples to measure the signals of out-of-plane electric field E_z of the samples. By analyzing the recorded field values, we obtain the distributions of the amplitude and phase of the out-of-plane electric field E_z.

Supplementary Note VI. Responses of microstrips to clockwise-phase-winding chiral source

The paramagnetic response of the structure with $M > 0$ under the excitation of +2π-phase winding chiral source is shown in Fig. 4 in the main text, where one can see that the Poynting vectors summed in individual unit cells rotate counterclockwise with respect to the sample center, which is the same as the chiral source. The measured and simulated local Poynting vectors under the excitation of −2π-phase winding chiral source are shown in Supplementary Fig. 6a and Supplementary Fig. 6b, respectively, which rotate clockwise in individual unit cells, same as the chiral source. The Poynting vectors summed in individual unit cell obtained by experiments and simulations shown in Supplementary Fig. 6c and Supplementary Fig. 6d, respectively, rotate

clockwise with respect to the system center, which is also same as the chiral source. Zoomed-in distributions of local Poynting vectors at the sample center under opposite chiral sources obtained by experiments and simulations are summarized in Supplementary Fig. 7, which demonstrates that the experimental results agree perfectly with simulations. Therefore, we can conclude that the structure with $M > 0$ responds to the chiral source in a paramagnetic way. Similarly, responses of the microstrip structure with $M < 0$ to chiral sources are summarized in Supplementary Fig. 8 and Supplementary Fig. 9. Surprisingly, while in individual unit cells the local Poynting vectors rotate in the same way as the chiral source, the Poynting vectors summed in individual unit cells rotate with respect to the sample center in a way opposite to the chiral source, which demonstrates the diamagnetic response of the structure with $M < 0$.

Supplementary Note VII. Relation between Poynting vector and Berry phase

Lemma 1: Suppose $\psi_{\mathbf{k}}(\mathbf{r})$ is a Bloch eigen wave function satisfying the master equation

$$-\frac{1}{\epsilon}\nabla^2\psi_{\mathbf{k}}(\mathbf{r}) = \frac{\omega^2}{c^2}\psi_{\mathbf{k}}(\mathbf{r}), \tag{A44}$$

with

$$\psi_{\mathbf{k}}(\mathbf{r}) = e^{i\mathbf{k}\cdot\mathbf{r}}u_{\mathbf{k}}(\mathbf{r}),$$

where $u_{\mathbf{k}}(\mathbf{r})$ is a periodic function with the same periodicity as the photonic crystal. Near the gapped Dirac cone at Γ point, one has

$$\iint_{\text{u.c.}}\psi_{\mathbf{k}}^*(\mathbf{r})\nabla\psi_{\mathbf{k}}(\mathbf{r})d\mathbf{r} = -\frac{\alpha}{2c^2}\iint_{\text{u.c.}} i\mathbf{k}\epsilon\psi_{\mathbf{k}}^*(\mathbf{r})\psi_{\mathbf{k}}(\mathbf{r})d\mathbf{r}, \tag{A45}$$

with the dispersion $\omega_{\mathbf{k}}^2 = \omega_{\text{edge}}^2 - \alpha\mathbf{k}^2/2$ for which $\alpha > 0$ below the band gap.

Proof: Writing $\psi_{\mathbf{k}}(\mathbf{r})$ in the form of Bloch wave $\psi_{\mathbf{k}}(\mathbf{r}) = e^{i\mathbf{k}\cdot\mathbf{r}}u_{\mathbf{k}}(\mathbf{r})$, the master equation (A44) becomes

$$\frac{1}{\epsilon}[-i\nabla + \mathbf{k}]^2 u_{\mathbf{k}}(\mathbf{r}) = \frac{\omega_{\mathbf{k}}^2}{c^2} u_{\mathbf{k}}(\mathbf{r}). \tag{A46}$$

For a small deviation $\mathbf{k}'$ from $\mathbf{k}$, one can expand Eq. (A46) as

$$\begin{aligned}\frac{1}{\epsilon}(-i\nabla + \mathbf{k})^2[u_{\mathbf{k}}(\mathbf{r}) + \mathbf{k}'\cdot\nabla_{\mathbf{k}}u_{\mathbf{k}}(\mathbf{r})] + 2\frac{1}{\epsilon}\mathbf{k}'\cdot(-i\nabla + \mathbf{k})u_{\mathbf{k}}(\mathbf{r}) + O\big(\mathbf{k}'^2\big) \\ = \frac{\omega_{\mathbf{k}}^2}{c^2}[u_{\mathbf{k}}(\mathbf{r}) + \mathbf{k}'\cdot\nabla_{\mathbf{k}}u_{\mathbf{k}}(\mathbf{r})] + \frac{1}{c^2}\mathbf{k}'\cdot(\nabla_{\mathbf{k}}\omega_{\mathbf{k}}^2)u_{\mathbf{k}}(\mathbf{r}) + O\big(\mathbf{k}'^2\big).\end{aligned} \tag{A47}$$

For the first order of $\mathbf{k}'$, one has

$$\begin{aligned}\frac{1}{\epsilon}(-i\nabla + \mathbf{k})^2[\mathbf{k}'\cdot\nabla_{\mathbf{k}}u_{\mathbf{k}}(\mathbf{r})] + 2\frac{1}{\epsilon}\mathbf{k}'\cdot(-i\nabla + \mathbf{k})u_{\mathbf{k}}(\mathbf{r}) \\ = \frac{\omega_{\mathbf{k}}^2}{c^2}\mathbf{k}'\cdot\nabla_{\mathbf{k}}u_{\mathbf{k}}(\mathbf{r}) + \frac{1}{c^2}\mathbf{k}'\cdot(\nabla_{\mathbf{k}}\omega_{\mathbf{k}}^2)u_{\mathbf{k}}(\mathbf{r}).\end{aligned} \tag{A48}$$

Noting that for the Hermitian operator $(-i\nabla + \mathbf{k})^2$ one has

$$\begin{aligned}&\iint_{\text{u.c.}} u_{\mathbf{k}}^*(\mathbf{r})(-i\nabla + \mathbf{k})^2[\mathbf{k}'\cdot\nabla_{\mathbf{k}}u_{\mathbf{k}}(\mathbf{r})]d\mathbf{r} \\ &= \iint_{\text{u.c.}} [(-i\nabla + \mathbf{k})^2 u_{\mathbf{k}}(\mathbf{r})]^*[\mathbf{k}'\cdot\nabla_{\mathbf{k}}u_{\mathbf{k}}(\mathbf{r})]d\mathbf{r} \\ &= \iint_{\text{u.c.}} \left[\frac{\omega_{\mathbf{k}}^2}{c^2}\epsilon u_{\mathbf{k}}(\mathbf{r})\right]^* [\mathbf{k}'\cdot\nabla_{\mathbf{k}}u_{\mathbf{k}}(\mathbf{r})]d\mathbf{r},\end{aligned}$$

we arrive at

$$\iint_{\text{u.c.}} u_{\mathbf{k}}^*(\mathbf{r})(-i\nabla + \mathbf{k})^2[\mathbf{k}'\cdot\nabla_{\mathbf{k}}u_{\mathbf{k}}(\mathbf{r})]d\mathbf{r} = \iint_{\text{u.c.}} \epsilon u_{\mathbf{k}}^*(\mathbf{r})\frac{\omega_{\mathbf{k}}^2}{c^2}[\mathbf{k}'\cdot\nabla_{\mathbf{k}}u_{\mathbf{k}}(\mathbf{r})]d\mathbf{r}. \tag{A49}$$

Integrating Eq. (A48) over the unit cell after multiplying $\epsilon u_{\mathbf{k}}^*(\mathbf{r})$ to the left of both sides, and using Eq. (A49), one obtains,

$$\iint_{\text{u.c.}} 2u_{\mathbf{k}}^*(\mathbf{r})\mathbf{k}'\cdot(-i\nabla+\mathbf{k})u_{\mathbf{k}}(\mathbf{r})d\mathbf{r} = \iint_{\text{u.c.}} \epsilon u_{\mathbf{k}}^*(\mathbf{r})\frac{1}{c^2}\mathbf{k}'\cdot(\nabla_{\mathbf{k}}\omega_{\mathbf{k}}^2)u_{\mathbf{k}}(\mathbf{r})d\mathbf{r},$$

$$\iint_{\text{u.c.}} -2iu_{\mathbf{k}}^*(\mathbf{r})e^{-i\mathbf{k}\cdot\mathbf{r}}e^{i\mathbf{k}\cdot\mathbf{r}}\mathbf{k}'\cdot(\nabla+i\mathbf{k})u_{\mathbf{k}}(\mathbf{r})d\mathbf{r} = \iint_{\text{u.c.}} \epsilon u_{\mathbf{k}}^*(\mathbf{r})e^{-i\mathbf{k}\cdot\mathbf{r}}e^{i\mathbf{k}\cdot\mathbf{r}}\frac{1}{c^2}\mathbf{k}'\cdot(\nabla_{\mathbf{k}}\omega_{\mathbf{k}}^2)u_{\mathbf{k}}(\mathbf{r})d\mathbf{r},$$

$$\mathbf{k}'\cdot\iint_{\text{u.c.}} -2i\psi_{\mathbf{k}}^*(\mathbf{r})\nabla\psi_{\mathbf{k}}(\mathbf{r})d\mathbf{r} = \mathbf{k}'\cdot\iint_{\text{u.c.}} \epsilon\frac{1}{c^2}(\nabla_{\mathbf{k}}\omega_{\mathbf{k}}^2)\psi_{\mathbf{k}}^*(\mathbf{r})\psi_{\mathbf{k}}(\mathbf{r})d\mathbf{r}. \quad \text{(A50)}$$

Eq. (A50) is satisfied for any small $\mathbf{k}'$, therefore,

$$\iint_{\text{u.c.}} -2i\psi_{\mathbf{k}}^*(\mathbf{r})\nabla\psi_{\mathbf{k}}(\mathbf{r})d\mathbf{r} = \iint_{\text{u.c.}} \epsilon\frac{1}{c^2}(\nabla_{\mathbf{k}}\omega_{\mathbf{k}}^2)\psi_{\mathbf{k}}^*(\mathbf{r})\psi_{\mathbf{k}}(\mathbf{r})d\mathbf{r},$$

$$\iint_{\text{u.c.}} \psi_{\mathbf{k}}^*(\mathbf{r})\nabla\psi_{\mathbf{k}}(\mathbf{r})\mathrm{d}\mathbf{r} = \iint_{\text{u.c.}} \frac{i}{2c^2}(\nabla_{\mathbf{k}}\omega_{\mathbf{k}}^2)\epsilon\psi_{\mathbf{k}}^*(\mathbf{r})\psi_{\mathbf{k}}(\mathbf{r})d\mathbf{r}. \quad \text{(A51)}$$

With the dispersion $\omega_{\mathbf{k}}^2 = \omega_{\text{edge}}^2 - \alpha\mathbf{k}^2/2$, one arrives at Eq. (A45). ■

The above Lemma applies to 2-dimensional photonic crystals with deformed honeycomb structure respecting $C_{6\text{v}}$ symmetry, with $\psi_{\boldsymbol{k}}$ standing for the out-of-plane electric field E_z/H_z of the TM/TE mode. It is also available for the spin-less electron with textured nearest-neighbor hopping energy on honeycomb structure[5].

Lemma 2: For modes in a PhC excited by a source characterized by circling Poynting vectors and placed at the central unit cell with frequency near the lower band edge around Γ point, one has

$$\mathbf{k}\times\mathbf{R}\psi_{\mathbf{k}}(\mathbf{R}+\mathbf{r}) = i\mathbf{k}\times\nabla_{\mathbf{k}}\psi_{\mathbf{k}}(\mathbf{R}+\mathbf{r}), \quad \text{(A52)}$$

with the position vectors shown in Supplementary Fig. 2.

Proof: The wavefunctions inside a unit cell which is far from the central unit cell are represented by the Bloch wave function $\psi_{\mathbf{k}}(\mathbf{R}+\mathbf{r})$, which satisfies the Bloch's theorem:

$$\psi_{\mathbf{k}}(\mathbf{R}+\mathbf{r}+\delta\mathbf{R}) = e^{i\mathbf{k}\cdot(\mathbf{R}+\mathbf{r}+\delta\mathbf{R})}u_{\mathbf{k}}(\mathbf{R}+\mathbf{r}) = e^{i\mathbf{k}\cdot\delta\mathbf{R}}\psi_{\mathbf{k}}(\mathbf{R}+\mathbf{r}). \quad \text{(A53)}$$

The excited modes have rotational symmetry around the central unit cell, one has

$$\psi_{\mathbf{k}}(\mathbf{r}+\mathbf{R}+\delta\mathbf{R}) = \psi_{\mathbf{k}+\delta\mathbf{k}}(\mathbf{R}+\mathbf{r}), \tag{A54}$$

where $\delta\mathbf{R}$ is the vector to a neighbor unit cell (see Supplementary Fig. 2). Using Bloch's theorem (A53) in the LHS of Eq. (A54) and expanding the RHS to the 1st order:

$$e^{i\mathbf{k}\cdot\delta\mathbf{R}}\psi_{\mathbf{k}}(\mathbf{R}+\mathbf{r}) = \psi_{\mathbf{k}}(\mathbf{R}+\mathbf{r}) + \delta\mathbf{k}\cdot\nabla_{\mathbf{k}}\psi_{\mathbf{k}}(\mathbf{R}+\mathbf{r}), \tag{A55}$$

Because $\mathbf{k}$ is small for frequency close to the band edge locating at the Γ point, we have $e^{i\mathbf{k}\cdot\delta\mathbf{R}} \simeq 1 + i\mathbf{k}\cdot\delta\mathbf{R}$. Eq. (A55) becomes

$$\begin{aligned}
i\mathbf{k}\cdot\delta\mathbf{R}\psi_{\mathbf{k}}(\mathbf{R}+\mathbf{r}) &= \delta\mathbf{k}\cdot\nabla_{\mathbf{k}}\psi_{\mathbf{k}}(\mathbf{R}+\mathbf{r}),\\
i\mathbf{k}\cdot(\delta\theta\mathbf{e}_z\times\mathbf{R})\psi_{\mathbf{k}}(\mathbf{R}+\mathbf{r}) &= (\delta\theta\mathbf{e}_z\times\mathbf{k})\cdot\nabla_{\mathbf{k}}\psi_{\mathbf{k}}(\mathbf{R}+\mathbf{r}),\\
-i\mathbf{e}_z\cdot(\mathbf{k}\times\mathbf{R})\psi_{\mathbf{k}}(\mathbf{R}+\mathbf{r}) &= \mathbf{e}_z\cdot[\mathbf{k}\times\nabla_{\mathbf{k}}\psi_{\mathbf{k}}(\mathbf{R}+\mathbf{r})],\\
-i\mathbf{k}\times\mathbf{R}\psi_{\mathbf{k}}(\mathbf{R}+\mathbf{r}) &= \mathbf{k}\times\nabla_{\mathbf{k}}\psi_{\mathbf{k}}(\mathbf{R}+\mathbf{r}),\\
\mathbf{k}\times\mathbf{R}\psi_{\mathbf{k}}(\mathbf{R}+\mathbf{r}) &= i\mathbf{k}\times\nabla_{\mathbf{k}}\psi_{\mathbf{k}}(\mathbf{R}+\mathbf{r}).
\end{aligned}$$

■

Derivation for the relation between Poynting vector and Berry phase

In order to see the response to an excitation source at the system center, we focus on a shell of unit cells which are at the radius $|\mathbf{R}| = R$ (See Fig. 2 in the main text). The time-averaged local Poynting vector for a TM mode is given by

$$\langle\mathbf{S}\rangle = \frac{1}{2\mu_0\omega}\mathrm{Im}\{E_z^*\nabla E_z\}, \tag{A56}$$

which yields an orbital angular momentum (OAM) with respect to the system center

$$\mathbf{L}_\mathrm{g}/\hbar = 2\mu_0\omega\sum_{|\mathbf{R}|=R}\mathbf{R}\times\iint_{u.c.}\langle\mathbf{S}\rangle d\mathbf{r}. \tag{A57}$$

Inserting Eq. (A56) to Eq. (A57), one has

$$\mathbf{L}_{\mathrm{g}}/\hbar = \sum_{|\mathbf{R}|=R} \mathrm{Im} \iint_{\mathrm{u.c.}} \mathbf{R} \times (E_z^* \nabla E_z) d\mathbf{r}.$$

The wavefunction E_z can be written as $E_z = f(|\mathbf{R}|)\psi_{\mathbf{k}}(\mathbf{R}+\mathbf{r})$, where $f(|\mathbf{R}|)$ is the real envelope function in the radial direction and $\psi_{\mathbf{k}}(\mathbf{R}+\mathbf{r})$ is the wavefunction normalized over a unit cell $\iint_{\mathrm{u.c.}} \epsilon \psi_{\mathbf{k}}^*(\mathbf{R}+\mathbf{r})\psi_{\mathbf{k}}(\mathbf{R}+\mathbf{r}) d\mathbf{r} = 1$, and $[f(|\mathbf{R}|)]^2 = \iint_{u.c.} \epsilon E_z^* E_z d\mathbf{r}$.

The OAM $\mathbf{L}_{\mathrm{g}}$ is evaluated as

$$\begin{aligned}
\mathbf{L}_{\mathrm{g}}/\hbar &= \sum_{|\mathbf{R}|=R} \mathrm{Im} \iint_{\mathrm{u.c.}} \mathbf{R} \times (E_z^* \nabla E_z) d\mathbf{r} \\
&= \sum_{|\mathbf{R}|=R} [f(|\mathbf{R}|)]^2 \mathrm{Im} \left[\mathbf{R} \times \iint_{\mathrm{u.c.}} (\psi_{\mathbf{k}}^* \nabla \psi_{\mathbf{k}}) d\mathbf{r}\right] \\
&= -\frac{\alpha}{2c^2} [f(R)]^2 \sum_{|\mathbf{R}|=R} \mathrm{Im} \iint_{\mathrm{u.c.}} \mathbf{R} \times i\mathbf{k} \epsilon \psi_{\mathbf{k}}^* \psi_{\mathbf{k}} d\mathbf{r} \\
&= \frac{\alpha}{2c^2} [f(R)]^2 \sum_{|\mathbf{R}|=R} \mathrm{Im} \iint_{\mathrm{u.c.}} i\epsilon \psi_{\mathbf{k}}^*(\mathbf{R}+\mathbf{r}) \mathbf{k} \times \mathbf{R} \psi_{\mathbf{k}}(\mathbf{R}+\mathbf{r}) d\mathbf{r} \\
&= \frac{\alpha}{2c^2} [f(R)]^2 \sum_{|\mathbf{R}|=R} \mathrm{Im} \iint_{\mathrm{u.c.}} i\epsilon \psi_{\mathbf{k}}^*(\mathbf{R}+\mathbf{r}) i\mathbf{k} \times \nabla_{\mathbf{k}} \psi_{\mathbf{k}}(\mathbf{R}+\mathbf{r}) d\mathbf{r} \\
&= -\frac{\alpha}{2c^2} [f(R)]^2 \sum_{|\mathbf{R}|=R} \mathbf{k} \times \mathrm{Im} \iint_{\mathrm{u.c.}} \epsilon \psi_{\mathbf{k}}^*(\mathbf{R}+\mathbf{r}) \nabla_{\mathbf{k}} \psi_{\mathbf{k}}(\mathbf{R}+\mathbf{r}) d\mathbf{r} \\
&= \frac{\alpha}{2c^2} [f(R)]^2 \sum_{|\mathbf{R}|=R} \mathbf{k} \times \boldsymbol{\mathcal{A}}_{\mathrm{n}}(\mathbf{k}),
\end{aligned}$$

where $\boldsymbol{\mathcal{A}}_{\mathrm{n}}(\mathbf{k}) = -\mathrm{Im} \iint_{\mathrm{u.c.}} \epsilon \psi_{\mathbf{k}}^*(\mathbf{R}+\mathbf{r}) \nabla_{\mathbf{k}} \psi_{\mathbf{k}}(\mathbf{R}+\mathbf{r}) d\mathbf{r}$ is the Berry connection. Replacing the summation note by integration $\sum_{|\mathbf{R}|=R} \rightarrow [1/(2\pi R)] \int R d\theta = [1/(2\pi)] \int d\theta$, which is a good approximation for $R \gg a_0$, one has

$$
\begin{aligned}
\mathbf{L}_{\mathrm{g}}/\hbar &= \frac{\alpha}{2c^2}[f(R)]^2\frac{1}{2\pi}\int \mathbf{k}\times\boldsymbol{\mathcal{A}}_{\mathrm{n}}(\mathbf{k})d\theta \\
&= \frac{\alpha}{4\pi c^2}\,[f(R)]^2\int d\theta\mathbf{k}\times\boldsymbol{\mathcal{A}}_{\mathrm{n}}(\mathbf{k}) \\
&= \frac{\alpha}{4\pi c^2}\,[f(R)]^2\int d\theta(k_R\mathbf{e}_R+k_\theta\mathbf{e}_\theta)\times\left[\mathcal{A}_{\mathrm{n}}^{\mathrm{R}}(\mathbf{k})\boldsymbol{e}_R+\mathcal{A}_{\mathrm{n}}^{\theta}(\mathbf{k})\boldsymbol{e}_\theta\right] \\
&= \mathbf{e}_z\frac{\alpha}{4\pi c^2}\,[f(R)]^2\int d\theta\left[-k_\theta\mathcal{A}_{\mathrm{n}}^{\mathrm{R}}(\mathbf{k})+k_R\mathcal{A}_{\mathrm{n}}^{\theta}(\mathbf{k})\right] \\
&= \mathbf{e}_z\frac{\alpha}{4\pi c^2}\,[f(R)]^2\int(-\mathbf{e}_Rk_\theta d\theta+\mathbf{e}_\theta k_R d\theta)\cdot\left[\mathbf{e}_R\mathcal{A}_{\mathrm{n}}^{\mathrm{R}}(\mathbf{k})+\mathbf{e}_\theta\mathcal{A}_{\mathrm{n}}^{\theta}(\mathbf{k})\right] \\
&= \mathbf{e}_z\frac{\alpha}{4\pi c^2}\,[f(R)]^2\oint_{|\mathbf{k}|=k}d\mathbf{k}\cdot\boldsymbol{\mathcal{A}}_{\mathrm{n}}(\mathbf{k}) \\
&= \mathbf{e}_z\frac{\alpha}{4\pi c^2}[f(R)]^2\,\gamma_n,
\end{aligned}
$$

where

$$
\gamma_n=\oint_{|\mathbf{k}|=k}d\mathbf{k}\cdot\boldsymbol{\mathcal{A}}_{\mathrm{n}}(\mathbf{k}) \tag{A58}
$$

is the Berry phase. Considering a cavity mode excited by a chiral source with positive phase winding, the value of γ_n is $-2\pi\le\gamma_n<0$ for $M<0$ and $0\le\gamma_n<2\pi$ for $M>0$ (see Supplementary Note 5). Therefore, $\mathbf{L}_g$ is parallel to the source for $M>0$, while for $M<0$, $\mathbf{L}_{\mathrm{g}}$ takes the opposite direction to the source.

Supplementary Note VIII. Berry curvatures in lumped-element LC circuit

Hamiltonian for the subspace spanned by $(|p_+\rangle,|d_+\rangle)^T$ is

$$\widehat{H}_+ = \omega_0^2 \begin{bmatrix} -(1-\tau) - \frac{1}{4}\tau a_0^2 k^2 & -\frac{i}{2}\tau a_0 k_+ \\ \frac{i}{2}\tau a_0 k_- & 1-\tau+\frac{1}{4}\tau a_0^2 k^2 \end{bmatrix}. \tag{A59}$$

Eigenmodes nearby the lower and upper band edges can be obtained by diagonalizing Hamiltonian (A59) as

$$|u_{\text{low}}\rangle = \frac{1}{\sqrt{N_{\text{low}}}} \begin{bmatrix} 2i\left(1-\tau+\frac{1}{4}\ \tau a_0^2 k^2\right) + 2i\sqrt{\frac{1}{4}\tau a_0^2 k^2 + \left(1-\tau+\frac{1}{4}\ \tau a_0^2 k^2\right)^2} \\ \tau a_0 k_- \end{bmatrix},$$

$$|u_{\text{up}}\rangle = \frac{1}{\sqrt{N_{\text{up}}}} \begin{bmatrix} 2i\left(1-\tau+\frac{1}{4}\ \tau a_0^2 k^2\right) - 2i\sqrt{\frac{1}{4}\tau a_0^2 k^2 + \left(1-\tau+\frac{1}{4}\ \tau a_0^2 k^2\right)^2} \\ \tau a_0 k_- \end{bmatrix}.$$

The Berry curvature is given by[6]

$$\Omega_{\text{low}}^+(k) \equiv i\,\frac{\langle u_{\text{low}}|\partial_{k_x}\widehat{H}_+|u_{\text{up}}\rangle\langle u_{\text{up}}|\partial_{k_y}\widehat{H}_+|u_{\text{low}}\rangle - \langle u_{\text{low}}|\partial_{k_y}\widehat{H}_+|u_{\text{up}}\rangle\langle u_{\text{up}}|\partial_{k_x}\widehat{H}_+|u_{\text{low}}\rangle}{4(t_0-t_1)^2}$$

$$= \frac{\tau^2 a_0^2\ \left(1-\tau-\frac{1}{4}\ \tau a_0^2 k^2\right)}{8\left[\frac{1}{4}\tau a_0^2 k^2 + \left(1-\tau+\frac{1}{4}\ \tau a_0^2 k^2\right)^2\right]^{3/2}}.$$

At Γ point $\Omega_{\text{low}}^+(k)$ takes the limit

$$\lim_{k\to 0}\Omega_{\text{n}}^+(k)\ = \frac{\tau^2 a_0^2}{8\,(1-\tau)^2}\,\text{Sgn}[1-\tau]. \tag{A60}$$

Equation (A60) indicates that for $M = (1-\tau)\omega_0^2 < 0$, one has $\lim_{k\to 0}\Omega_{\text{n}}^+(k) < 0$, whereas for $M = (1-\tau)\omega_0^2 > 0$, one has $\lim_{k\to 0}\Omega_{\text{n}}^+(k) > 0$.

The Berry curvature $\Omega_{\text{low}}^+(k)$ integrated over the whole k space is

$$\int_0^{2\pi} d\theta \int_0^{\infty} \Omega_{\text{low}}^{+}(k) k dk = \begin{cases} -2\pi, & \tau > 1; \\ 0, & \tau < 1. \end{cases}$$

Supplementary Note IX. OAM of the excited modes

The Poynting vectors integrated over individual unit cells shown in Fig. 4 and Fig. 5 of the main text for the modes excited by the sources with positive chirality, and Supplementary Fig. 6, Supplementary Fig. 8 for the modes excited by the sources with negative chirality can be translated into OAM defined by Eq. (A57). For $M > 0$ structure excited by a +2π-phase winding chiral source, the measured and simulated OAM of each unit cell are displayed in Supplementary Fig. 10a and Supplementary Fig. 10b by the numbers inside the yellow dashed hexagons, respectively. Both experimental and simulation results show positive OAM which are same as the chiral source. The measured and simulated OAM for the mode excited by a -2π-phase winding source are shown in Supplementary Fig. 11a and Supplementary Fig. 11b, which both have negative sign, same as the chiral source. For $M < 0$ structure excited by a $\pm$4π-phase winding source, the results are shown in Supplementary Fig. 12a and Supplementary Fig. 12b and Supplementary Fig. 13a and Supplementary Fig. 13b, where the OAM of individual unit cells have the opposite sign to the chiral source.

There is a stricter definition for the global OAM with respect to the cavity center, where the integration is after the vector multiplication of local Poynting vectors and their positions, $\mathbf{L}_{\text{g}}/\hbar = 2\mu_0\omega \sum_{\mathbf{R}} \iint_{\text{u.c.}} (\mathbf{R}+\mathbf{r}) \times \langle \mathbf{S} \rangle d\mathbf{r}$. The results are shown in subplots c and d of Supplementary Fig. 10, Supplementary Fig. 11, Supplementary Fig. 12 and Supplementary Fig. 13, where the signs of OAM are same to those in subplots a and b. The difference of the values under the two definitions

in the same unit cell is the OAM of that unit cell with respect to its own center, whose sign is determined by the chirality of the excited mode, despite the sign of the Dirac mass M. This result indicates that the diamagnetic (paramagnetic) chiral response for $M < 0$ ($M > 0$) can be satisfied even when the chirality-determined term is considered.

Supplementary Note X. Chiral response of the microstrip structures without cladding

The paramagnetic and diamagnetic response of the structures $M > 0$ and $M < 0$ shown in Figs. 4 and 5 of the main text are cladded by dual structures with $M < 0$ and $M > 0$. In fact, it should be emphasized that the cladding layer is indeed not a must for observing the novel chiral response. Considering the chiral source with counterclockwise phase winding, the full-wave computer simulated chiral response of large pure microstrip structures of $M > 0$ and $M < 0$ with 127 C_{6v}-symmetric unit cells exposing to air are shown in Supplementary Fig. 14 and Supplementary Fig. 15, respectively. From the precise amplitude and phase of the out-of-plane electric field E_z, the local Poynting vector can be mapped out, as shown in Supplementary Fig. 14d and Supplementary Fig. 15d. Especially, for $M > 0$ and $M < 0$ structures, the local Poynting vectors in individual unit cells rotate counterclockwise, which is the same as the chiral source. However, the Poynting vectors summed in individual C_{6v}-symmetric unit cells rotate counterclockwise and clockwise with respect to the center of the $M > 0$ and $M < 0$ structures; so pure microstrip structures of $M > 0$ and $M < 0$ exhibit the paramagnetic and diamagnetic chiral response, respectively. Same as Supplementary Fig. 14 and Supplementary Fig. 15, Supplementary Fig. 16 and Supplementary Fig. 17 show the chiral response correspondence in the microstrip structures without cladding under the excitation of clockwise phase winding chiral source. Therefore, the chiral response remains

unchanged in the two topologically distinct microstrip structures when cladding layers of dual microstrip structures are removed.

Supplementary Note XI. Chiral response for source located at the C_{3v}-symmetric area

In the main text, we discuss on the chiral response when the chiral source is located in C_{6v}-symmetric unit cell since it is easy to achieve in experiments with frequency set slightly below the lower band edge. In principle, one can shift the chiral source to the C_{3v}-symmetric area among three C_{6v}-symmetric unit cells, and shift the frequency to the band gap. From full-wave simulations we find that the chiral response depends crucially on whether the frequency is set in the passband or falls in the bandgap.

Case 1*: *frequency in passband

The phenomenology remains unchanged when the source is shifted to the C_{3v}-symmetric area among three C_{6v}-symmetric unit cells: the global bulk EM flow is opposite to the chiral source in the structure with $M < 0$ whereas it is the same in the structure with $M > 0$. Take the counterclockwise phase winding chiral source for example, the full-wave computer simulated distribution of local Poynting vectors of large pure microstrip structures with $M > 0$ and $M < 0$ with 108 C_{6v}-symmetric unit cells exposing to air are shown in Supplementary Fig. 18a and Supplementary Fig. 18d, respectively. The counterclockwise pseudospin state in all C_{6v}-symmetric unit cells (marked by the dashed black lines) is the same as the counterclockwise chiral source as shown in Supplementary Fig. 18b and Supplementary Fig. 18e for $M > 0$ and $M < 0$, respectively. Supplementary Fig. 18c and Supplementary Fig. 18f show that the Poynting vectors summed in individual C_{6v}-symmetric unit cells rotate counterclockwise (clockwise) with respect to the center

of $M > 0$ ($M < 0$) structure, parallel (opposite) to the source, corresponding to the paramagnetic (diamagnetic) chiral response. Similar results but for clockwise phase winding are shown in Supplementary Fig. 19.

As can be read from Figs S18 and S19, for a passband frequency under concern the whirling direction of local Poynting vectors in C_{6v}-symmetric unit cells, which specifies the pseudospin state, is the same as the chiral source, irrespective to the location of the chiral source, either in the C_{6v}-symmetric unit cells or in the C_{3v}-symmetric area among three C_{6v}-symmetric unit cells.

Case 2*: *frequency in band gap

For in-gap frequency, the whirling direction of local Poynting vectors in C_{6v}-symmetric unit cells is the same as the chiral source only when the chiral source is located in a C_{6v}-symmetric unit cell, as shown in Supplementary Fig. 20a and Supplementary Fig. 20b. On the contrary, the whirling direction of local Poynting vectors in C_{6v}-symmetric unit cells is opposite to the chiral source when the source is located in the C_{3v}-symmetric area as shown in Supplementary Fig. 20c and Supplementary Fig. 20d. Similar to Supplementary Fig. 20, Supplementary Fig. 21 shows the simulated chiral response under the excitation by a chiral source with clockwise phase winding.

Therefore, the location of chiral source, i.e. in a C_{6v}-symmetric unit cell and/or in the C_{3v}-symmetric area among unit cells, leaves different impacts on the mode excitation when the frequency is set in passband and/or falls in bandgap.

Supplementary Note XII. Negative refraction and left-handedness

It has been revealed that a photonic crystal with an upwardly convex frequency dispersion below the band gap at Γ point is characterized by a negative refractive index and left-handedness[7-9]. For

the present system, we have $\omega_{\mathbf{k}}^2 = (2 + \tau - |M|)\omega_0^2 - \alpha\mathbf{k}^2/2$ slightly below the lower band edge with $\alpha > 0$ as given in Eq. (A5), which indicates that the Poynting vector (same as the group velocity) is antiparallel to the Bloch wave number $\mathbf{k}$. Therefore, the system behaves left-handedly irrespective to the Dirac mass sign. A photonic crystal formed by puncturing triangle airholes in a dielectric material[3,10] can be used to demonstrate the negative refraction as shown in Supplementary Fig. 22, where the photonic crystal is trivial with $M > 0$ for $a_0 > 3R$ and topological with $M < 0$ for $a_0 > 3R$. In simulations performed by using COMSOL Multiphysics[11], we consider a beam of plane wave incident to a ribbon of photonic crystal with 20 $C_{6\mathrm{v}}$-symmetric unit cells in width and the frequency is set slightly below the lower band edge. Negative refraction is observed in both trivial and topological systems as shown in Supplementary Fig. 22d and Supplementary Fig. 22e. Above the band gap, one has $\omega_{\mathbf{k}}^2 = (2 + \tau + |M|)\omega_0^2 + \alpha\mathbf{k}^2/2$. The Poynting vector is thus parallel to the Bloch wave number, and the system is right-handed. The four cases of diamagnetic/paramagnetic chiral response and left-/right-handedness are summarized in Supplementary Fig. 23.

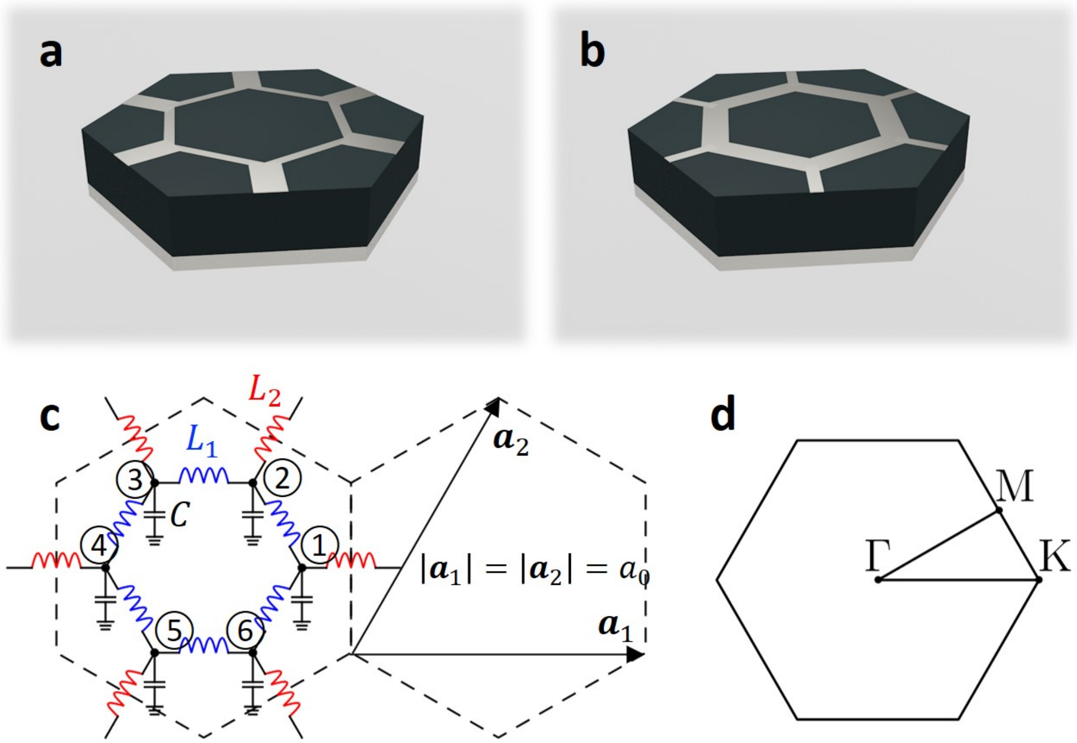

Supplementary Fig. 1. (**a**) and (**b**), Schematic of the hexagonal unit cell of honeycomb microstrip with $M < 0$ and $M > 0$. (**c**), Lumped-element LC circuit. All nodes are connected individually to the common ground via capacitors of uniform capacitance C, and are connected to three nearest-neighbor nodes by inductors with inductance L_1 (blue color) inside unit cells and L_2 (red color) between $C_{6\mathrm{v}}$-symmetric unit cells. (**d**), The first Brillouin zone corresponding to the $C_{6\mathrm{v}}$-symmetric unit cell in (**c**).

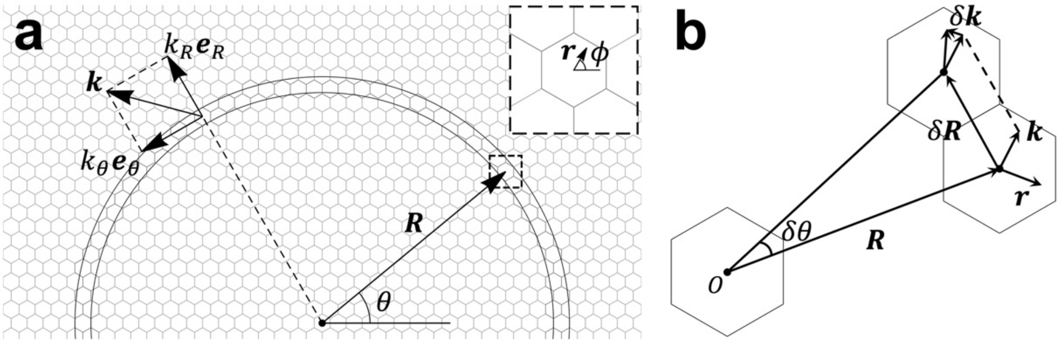

Supplementary Fig. 2. (**a**) Schematic of the $C_{6\mathrm{v}}$-symmetric unit cells located in a shell at $|\mathbf{R}| = R \gg a_0$. **R** represents the position of the center of the unit cell and **r** means the position inside the unit cell. The wavefunction inside the unit cell is described by Bloch wavefunction $u_{\mathbf{k}}(\mathbf{R}+\mathbf{r})$. (**b**) Two neighboring unit cells in the shell shown in (**a**).

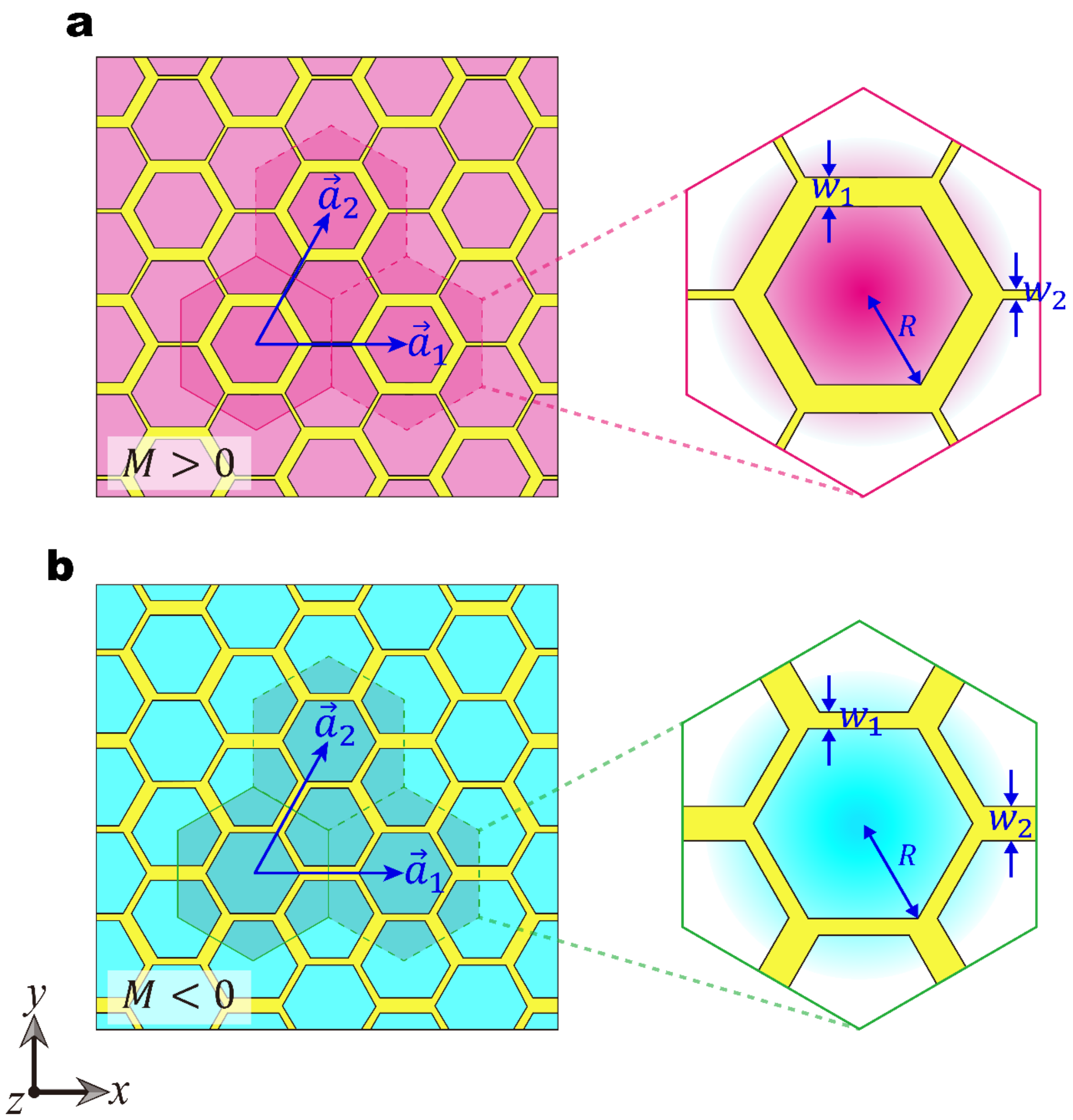

Supplementary Fig. 3. (**a**) and (**b**) Schematic of the honeycomb microstrip-based structures with $\boldsymbol{M} > 0$ and $\boldsymbol{M} < 0$. The two unit vectors are indicated by blue arrows. Insets show zoomed-in $\boldsymbol{C_{6v}}$-symmetric unit cells of the microstrip-based structures. For $\boldsymbol{M} > 0$, the structure parameters are $\boldsymbol{w_1}$= 2.6 mm, $\boldsymbol{w_2}$ = 0.9 mm and $\boldsymbol{R}$ = 9.4 mm, whereas for $\boldsymbol{M} < 0$, $\boldsymbol{w_1}$= 1.5 mm, $\boldsymbol{w_2}$ = 3.2 mm and $\boldsymbol{R}$ = 10 mm, respectively. For both structures $\boldsymbol{a_1} = \boldsymbol{a_2}$ = 32.6mm.

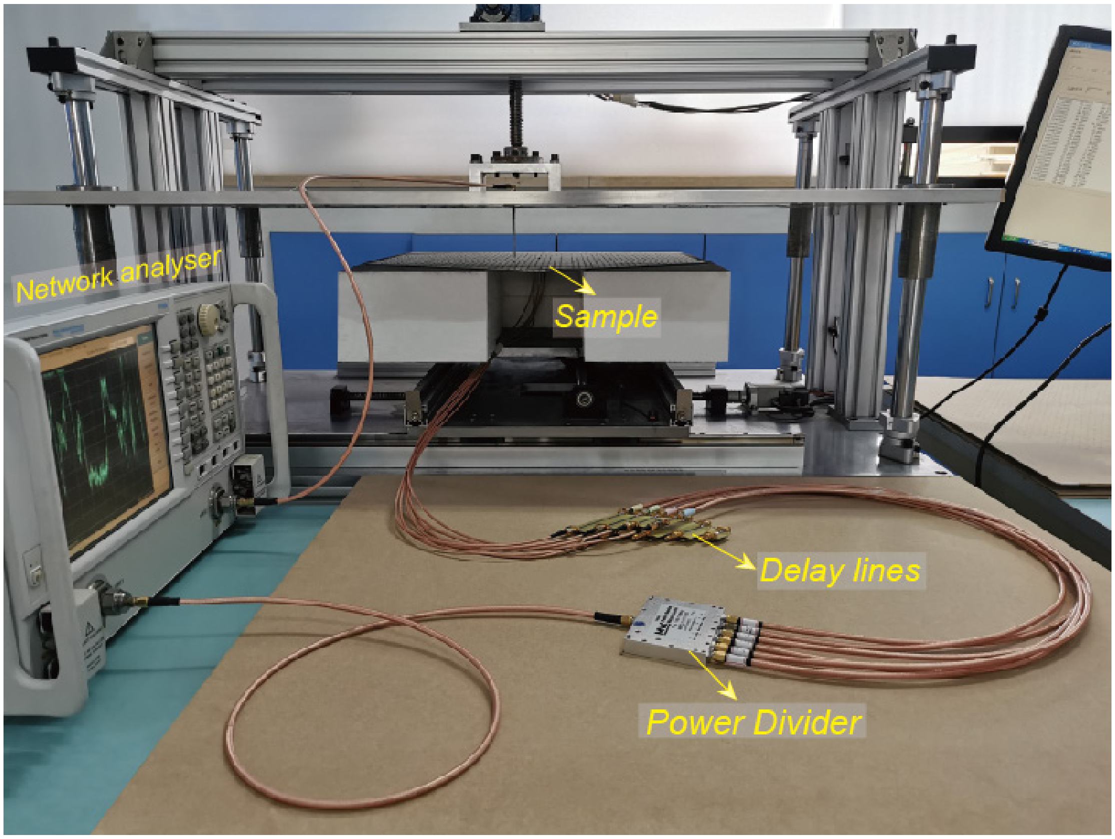

Supplementary Fig. 4. Photo and diagram of experimental setup. The experimental setup is composed of a vector network analyzer, a 2D translational stage, a power divider, delay lines and the sample to be measured. The sample is put on a 10-cm-thick foam substrate with a permittivity of near one. The signals are picked up by the small homemade rod antenna (electric probe) mounted in the translational stage above the planar structure, which are used to measure the amplitude and phase distributions of the out-of-plane electric field $\mathbf{E_z}$.

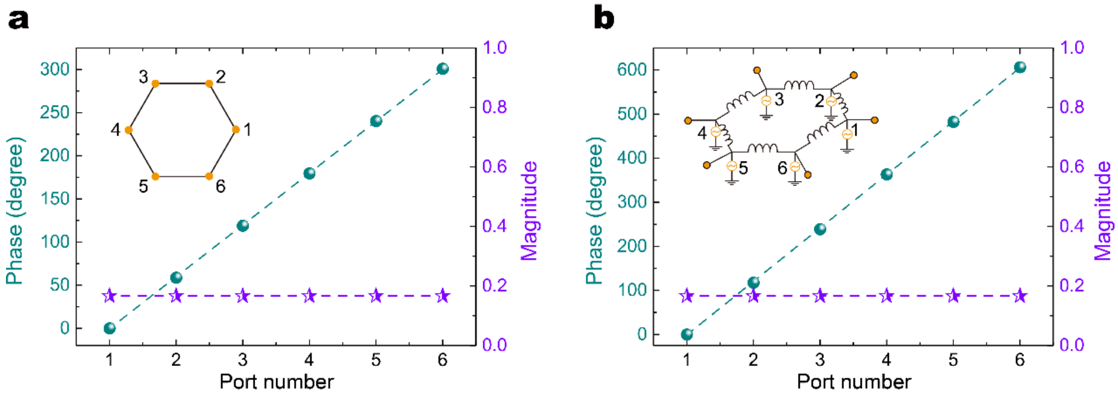

Supplementary Fig. 5. (**a**) and (**b**) Experimental realization of two kinds of chiral sources of $+2\pi$- and $+4\pi$-phase winding, where the relative phase shift between six ports is controlled to 60 degrees and 120 degrees, respectively. The transmission magnitudes of all six ports for two kinds of chiral sources are the same. Inset in (**a**) shows the design principle of chiral source where a port array with phase delays is used. Inset in (**b**) indicates the corresponding LC circuit model of the chiral source in microstrip structure.

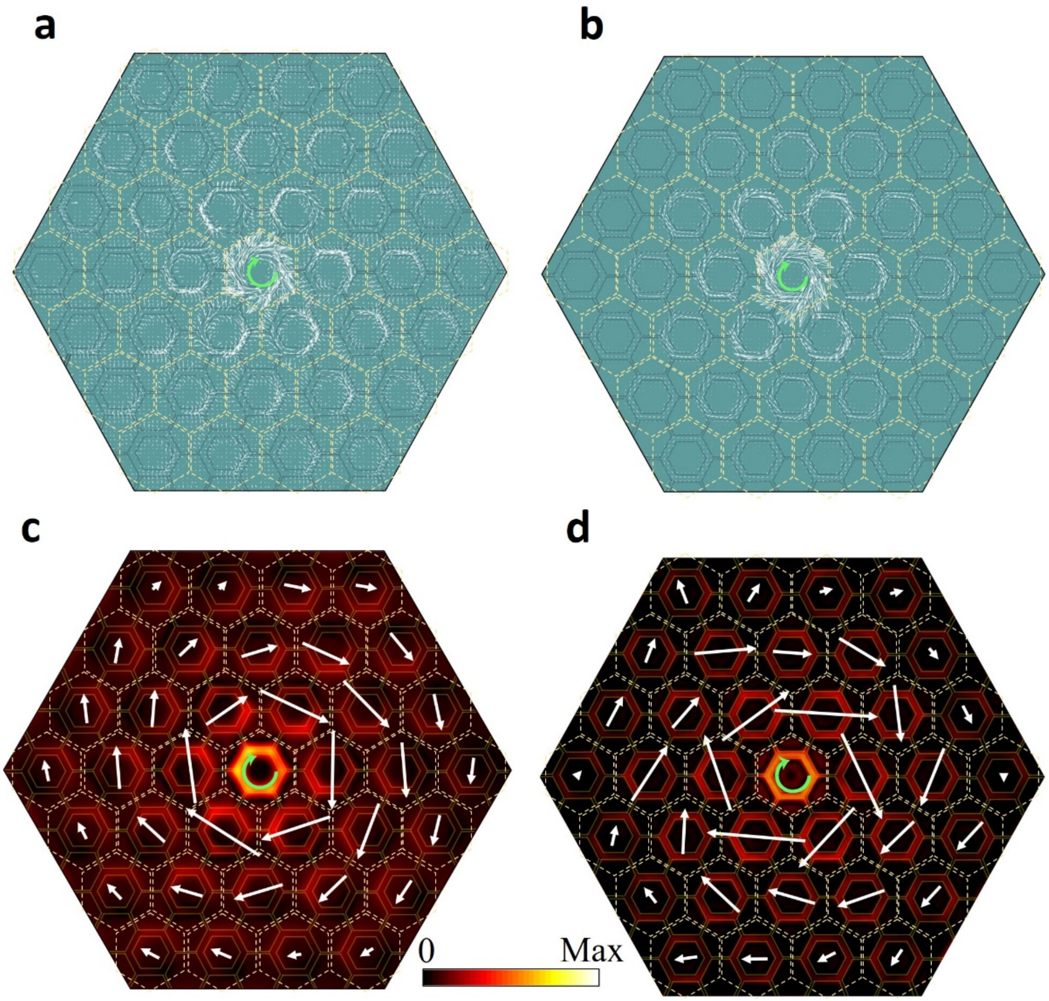

Supplementary Fig. 6. (**a**) and (**b**) Distributions of local Poynting vectors obtained by experiments at 1.43 GHz and by simulations at 1.42 GHz, respectively, for microstrip structure with $M > 0$ under the excitation of -2π-phase winding chiral source. The local Poynting vectors in individual $C_{6\mathrm{v}}$-symmetric unit cells rotate clockwise, which is the same as the chiral source. (**c**) and (**d**) Poynting vectors summed in individual unit cells obtained by experiments and simulations, respectively, rotate clockwise with respect to the system center, which is the same as the chiral source. These indicate that the microstrip structure with $M > 0$ responds to the chiral source in a paramagnetic way.

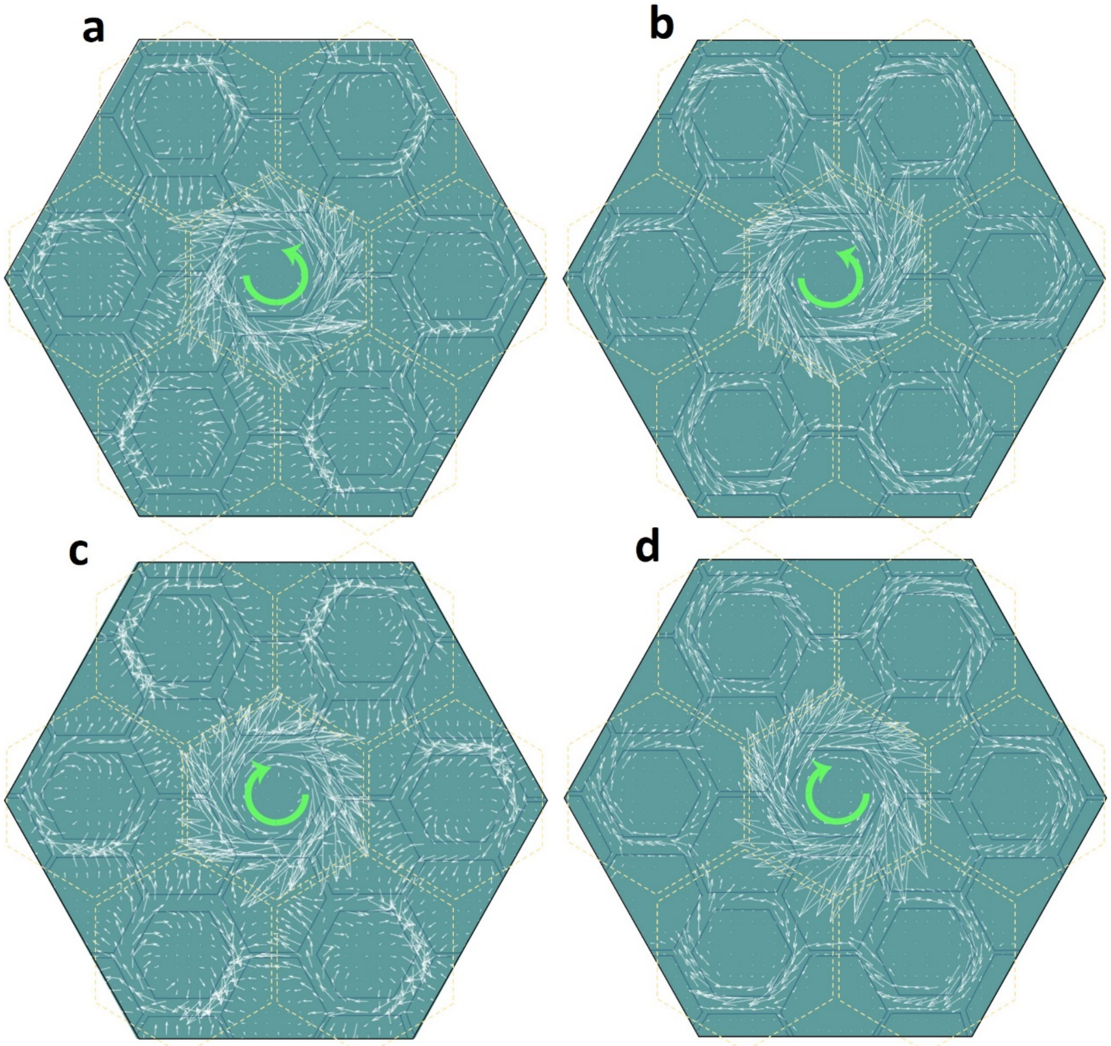

Supplementary Fig. 7. (**a**) and (**c**) Zoomed-in view of the measured local Poynting vectors in the $M > 0$ structure at 1.43 GHz under the excitation of +2π- and -2π-phase winding chiral source, respectively. (**b**) and (**d**), Same as (**a**) and (**c**) except obtained by the full-wave simulations at 1.42 GHz. These indicate the time-reversal symmetry of the present system.

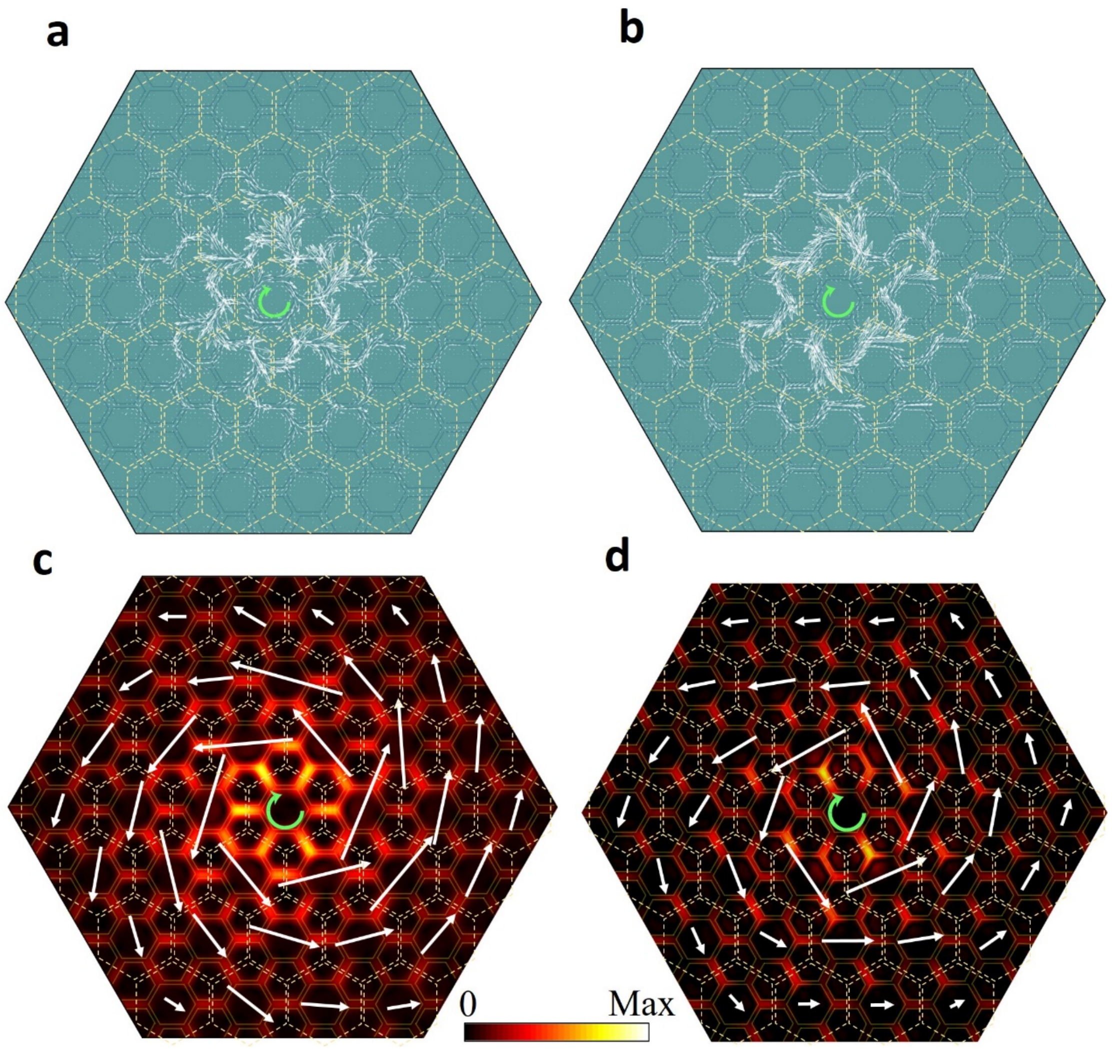

Supplementary Fig. 8. (**a**) and (**b**) Distributions of local Poynting vectors obtained by experiments at 1.445 GHz and by simulations at 1.42 GHz, respectively, for microstrip structure with $M < 0$ under the excitation of -4π-phase winding chiral source. The local Poynting vectors in individual $C_{6\mathrm{v}}$-symmetric unit cells rotate clockwise, which is the same as the chiral source. (**c**) and (**d**) Poynting vectors summed in individual unit cells obtained by experiments and simulations, respectively, rotate counterclockwise with respect to the system center, which is opposite to the chiral source. These indicate that the microstrip structure with $M < 0$ responds to the chiral source in a diamagnetic way.

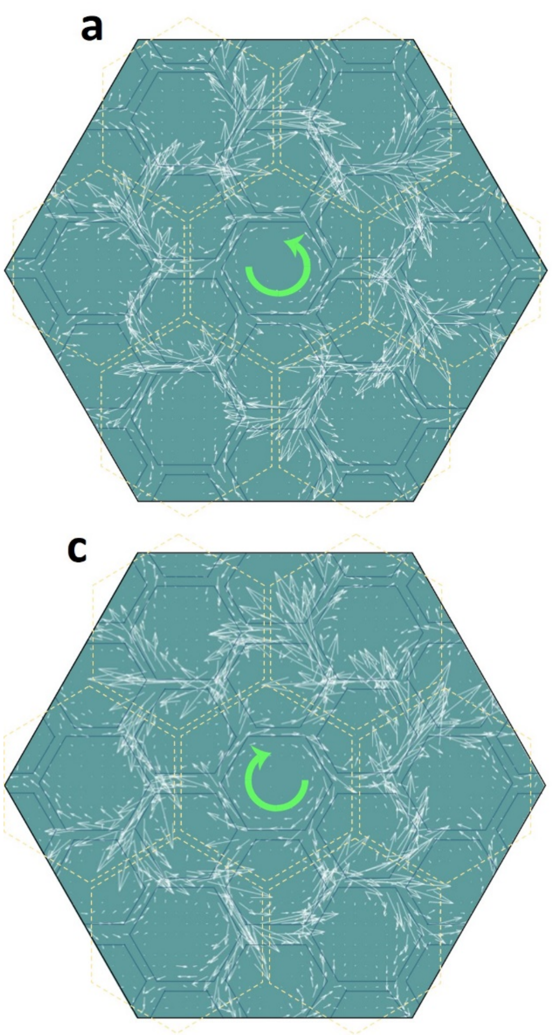

Supplementary Fig. 9. (**a**) and (**c**) Zoomed-in view of the local Poynting vectors in the $M < 0$ structure at 1.445 GHz under the excitation of +4π-phase and -4π-phase winding chiral source, respectively. (**b**) and (**d**) Same as (**a**) and (**c**) except obtained by the full-wave simulations at 1.42 GHz. These indicate the time-reversal symmetry of the present system.

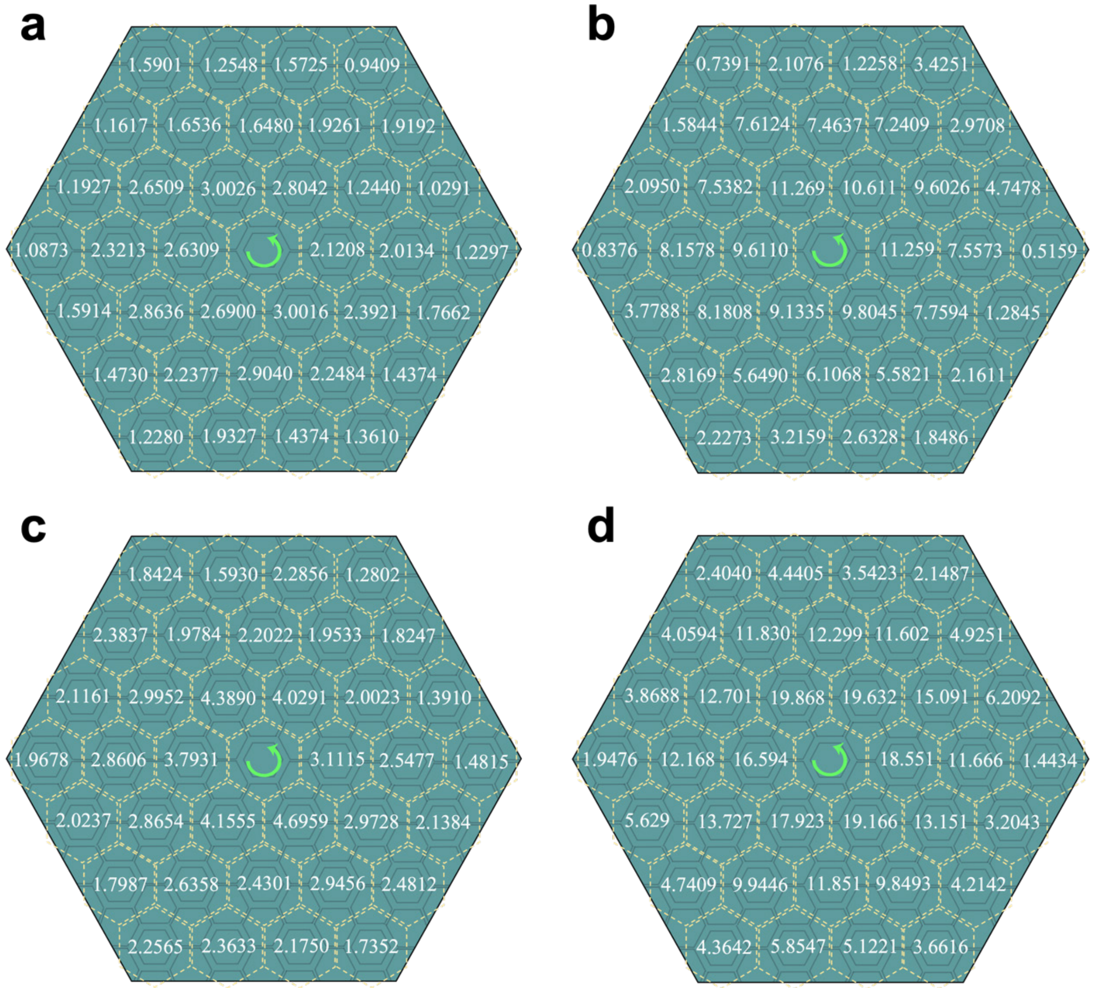

Supplementary Fig. 10. (**a**) and (**b**) Orbital angular momenta (OAM) of each $C_{6\mathrm{v}}$-symmetric unit cells (white numbers), defined by Eq. (A57), with respect to the center of the cavity for the $M > 0$ structure under the excitation of 2π-winding source obtained by experiment and simulation, respectively. Note that the Poynting vectors are integrated first in individual $C_{6\mathrm{v}}$-symmetric unit cells. (**c**) and (**d**) Experimentally and numerically obtained results for local OAM with respect to the cavity center integrated over individual $C_{6\mathrm{v}}$-symmetric unit cells for the $M > 0$ structure under the excitation of 2π-winding source.

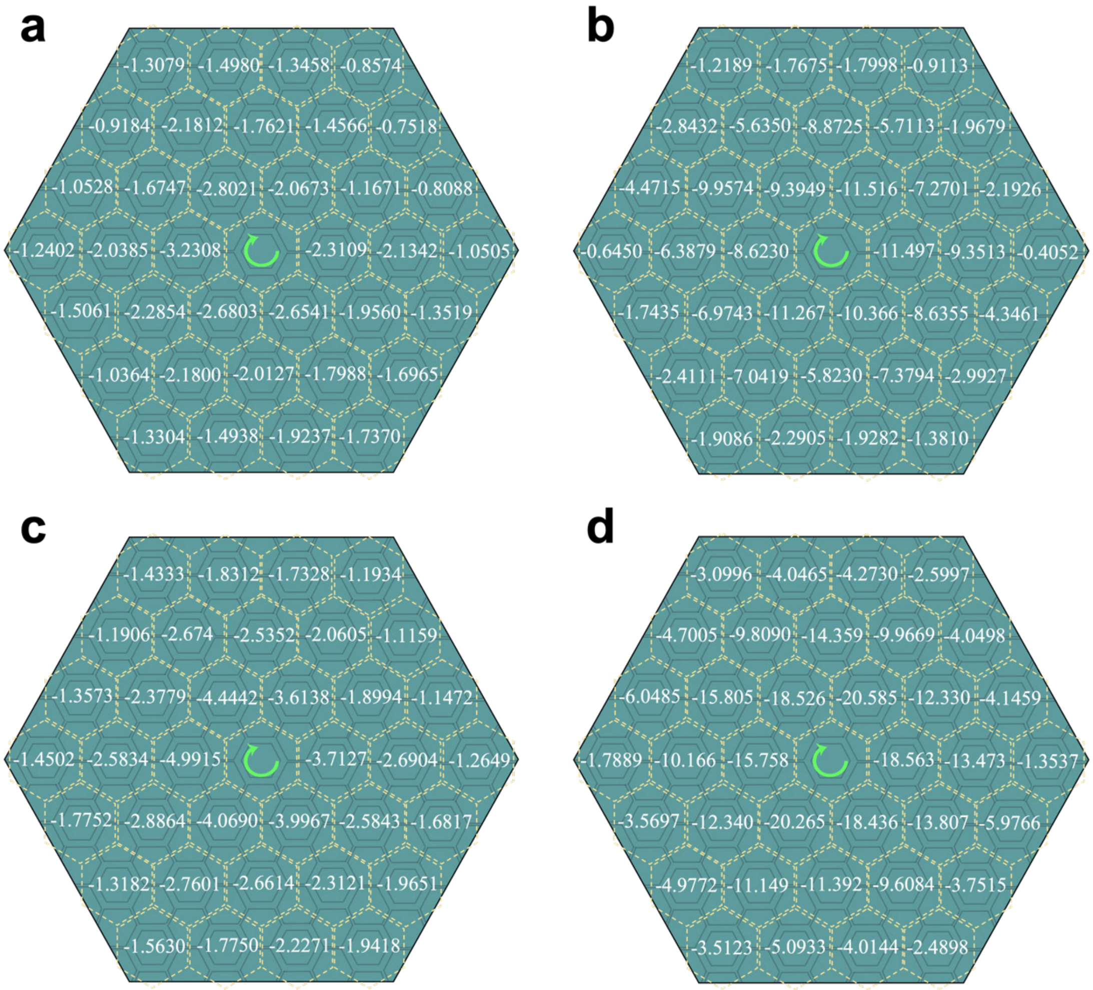

Supplementary Fig. 11. (**a**) and (**b**) Orbital angular momenta (OAM) of each C_{6v}-symmetric unit cells (white numbers), defined by Eq. (A57), with respect to the center of the cavity for the $M > 0$ structure under the excitation of -2π-winding source obtained by experiment and simulation, respectively. Note that the Poynting vectors are integrated first in individual C_{6v}-symmetric unit cells. (**c**) and (**d**) Experimentally and numerically obtained results for local OAM with respect to the cavity center integrated over individual C_{6v}-symmetric unit cells for the $M > 0$ structure under the excitation of -2π-winding source.

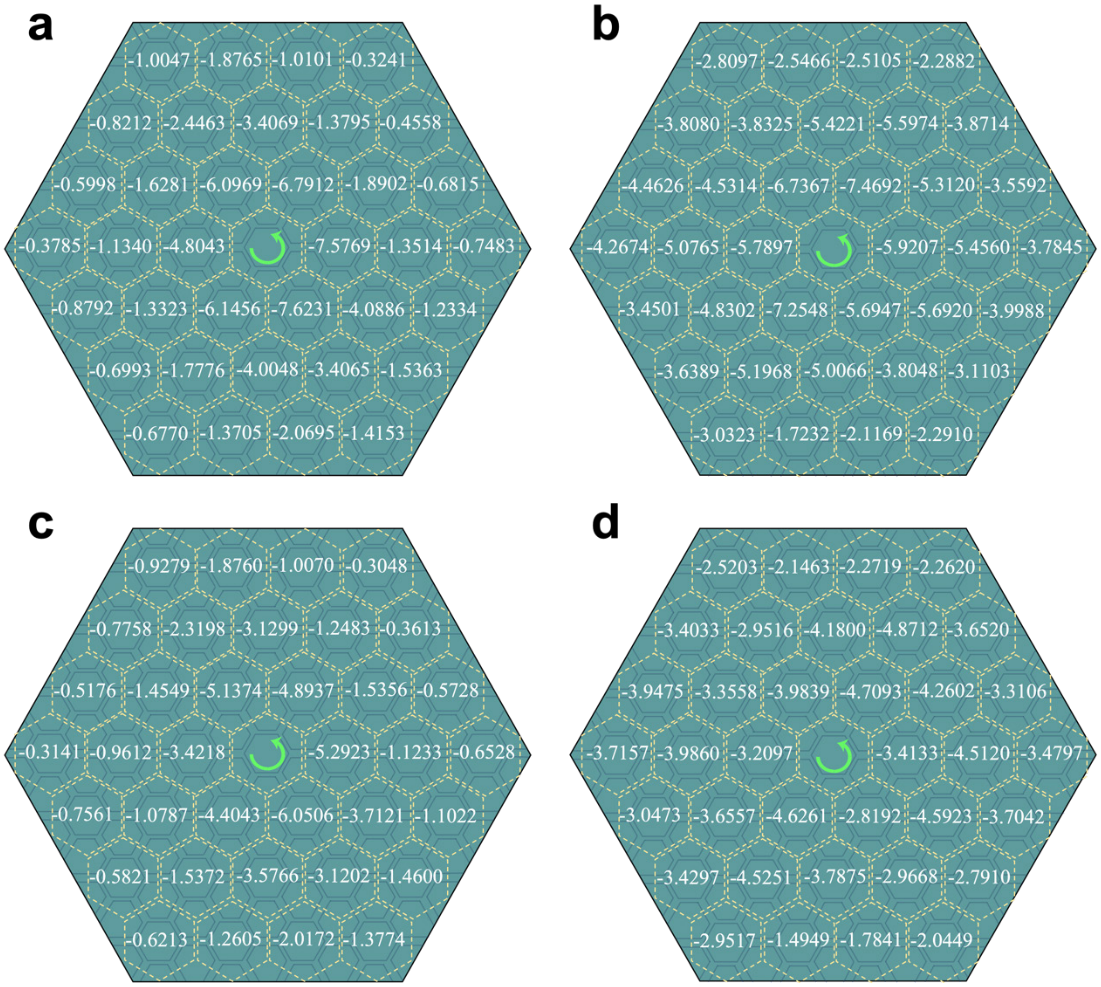

Supplementary Fig. 12. (**a**) and (**b**) Orbital angular momenta (OAM) of each $C_{6\mathrm{v}}$-symmetric unit cells (white numbers), defined by Eq. (A57), with respect to the center of the cavity for the $M < 0$ structure under the excitation of 4π winding source obtained by experiment and simulation, respectively. Note that the Poynting vectors are integrated first in individual $C_{6\mathrm{v}}$-symmetric unit cells. (**c**) and (**d**) Experimentally and numerically obtained results for local OAM with respect to the cavity center integrated over individual $C_{6\mathrm{v}}$-symmetric unit cells for the $M < 0$ structure under the excitation of 4π-winding source.

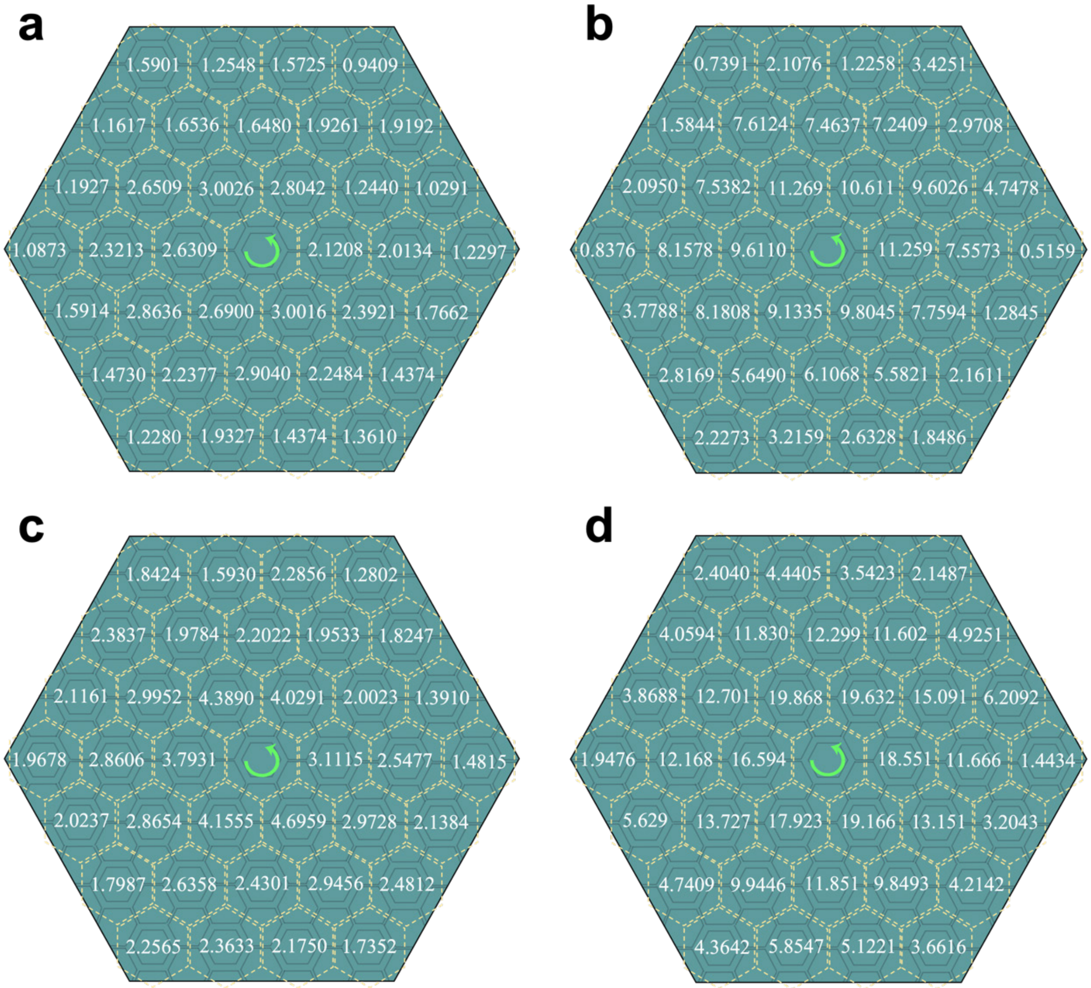

Supplementary Fig. 13. (**a**) and (**b**) Orbital angular momenta (OAM) of each $C_{6\mathrm{v}}$-symmetric unit cells (white numbers), defined by Eq. (A57), with respect to the center of the cavity for the $M < 0$ structure under the excitation of -4π-winding source obtained by experiment and simulation, respectively. Note that the Poynting vectors are integrated first in individual $C_{6\mathrm{v}}$-symmetric unit cells. (**c**) and (**d**) Experimentally and numerically obtained results for local OAM with respect to the cavity center integrated over individual $C_{6\mathrm{v}}$-symmetric unit cells for the $M < 0$ structure under the excitation of -4π-winding source.

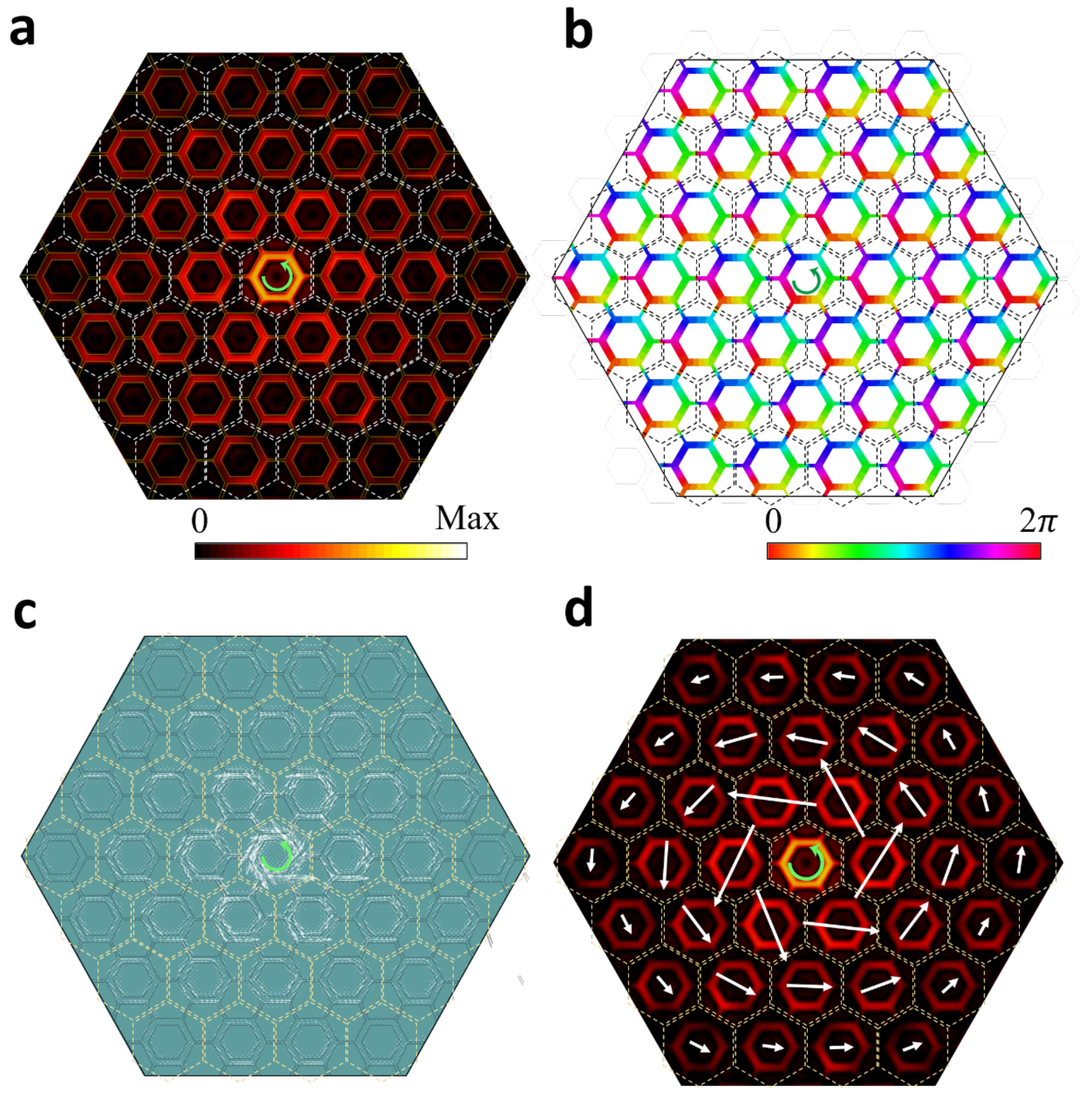

Supplementary Fig. 14. Chiral response of the $M > 0$ structure without cladding to a chiral source with counterclockwise phase winding. (**a**) Distribution of the strength of out-of-plane electric-field E_z in the central part of the structure, which is obtained by the full-wave simulations using a +2π-phase-winding chiral source located in a $C_{6\mathrm{v}}$-symmetric unit cell at the sample center at f = 1.42 GHz. (**b**) Distribution of the phase of the out-of-plane electric-field E_z under the same condition as (**a**). (**c**) Distribution of local Poynting vectors obtained by the amplitude (**a**) and phase (**b**) of the out-of-plane electric-field E_z. (**d**) Distribution of Poynting vectors summed in individual $C_{6\mathrm{v}}$-symmetric unit cells obtained from (**c**). The local Poynting vectors in individual $C_{6\mathrm{v}}$-symmetric

unit cells rotate counterclockwise, which is the same as the chiral source. The Poynting vectors summed in individual $C_{6\mathrm{v}}$-symmetric unit cells rotate counterclockwise with respect to the sample center, same to the source, corresponding to the paramagnetic chiral response.

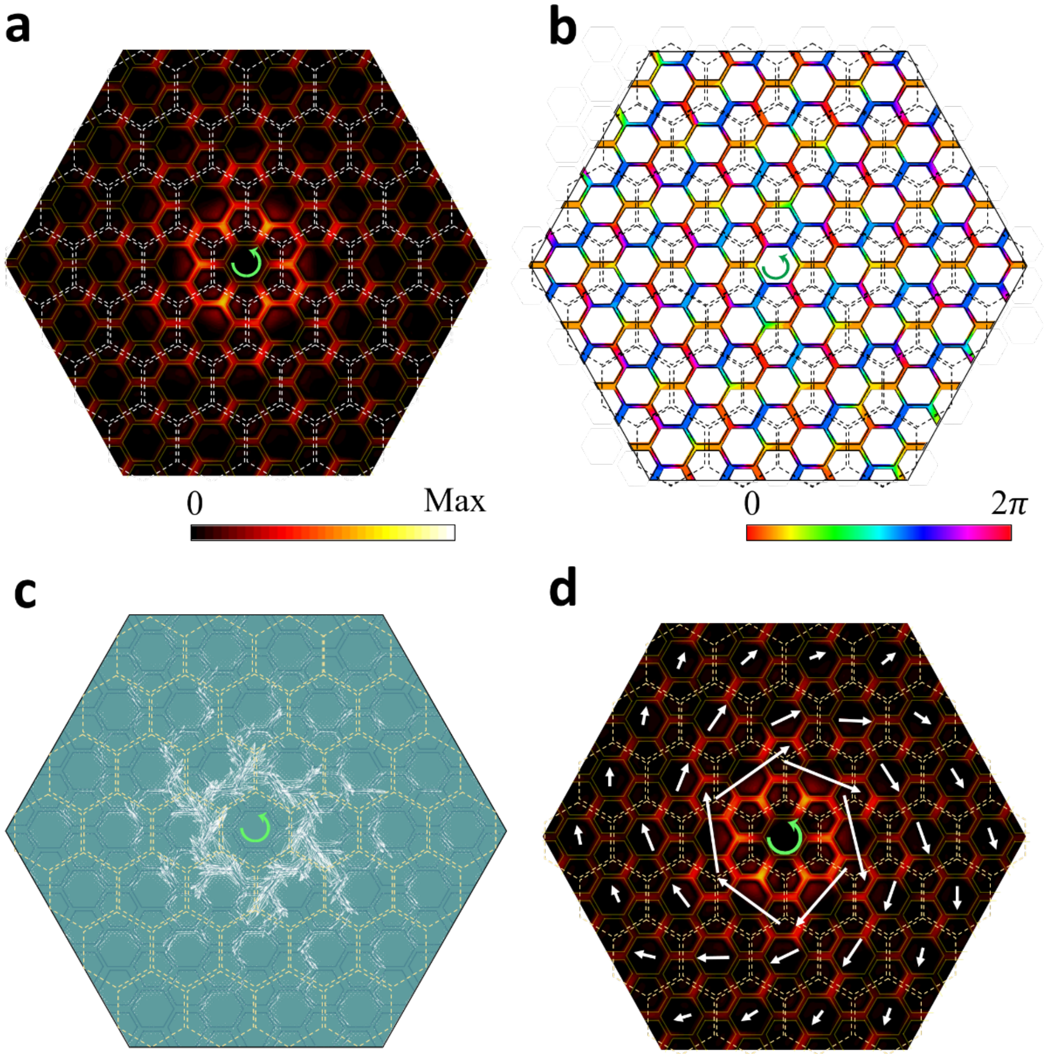

Supplementary Fig. 15. Chiral response of the $M < 0$ structure without cladding to a chiral source with counterclockwise phase winding. (**a**) Distribution of the strength of out-of-plane electric-field E_z in the central part of the structure, which is obtained by the full-wave simulations using a +4π-phase-winding chiral source located in a C_{6v}-symmetric unit cell at the sample center at f = 1.42 GHz. (**b**) Distribution of the phase of the out-of-plane electric-field E_z under the same condition as (**a**). (**c**) Distribution of local Poynting vectors obtained by the amplitude (**a**) and phase (**b**) of the out-of-plane electric-field E_z. (**d**) Distribution of Poynting vectors summed in individual C_{6v}-symmetric unit cells obtained from (**c**). The local Poynting vectors in individual C_{6v}-

symmetric unit cells rotate counterclockwise, which is the same as the chiral source. The Poynting vectors summed in individual $C_{6\mathrm{v}}$-symmetric unit cells rotate clockwise with respect to the sample center, opposite to the source, corresponding to the diamagnetic chiral response.

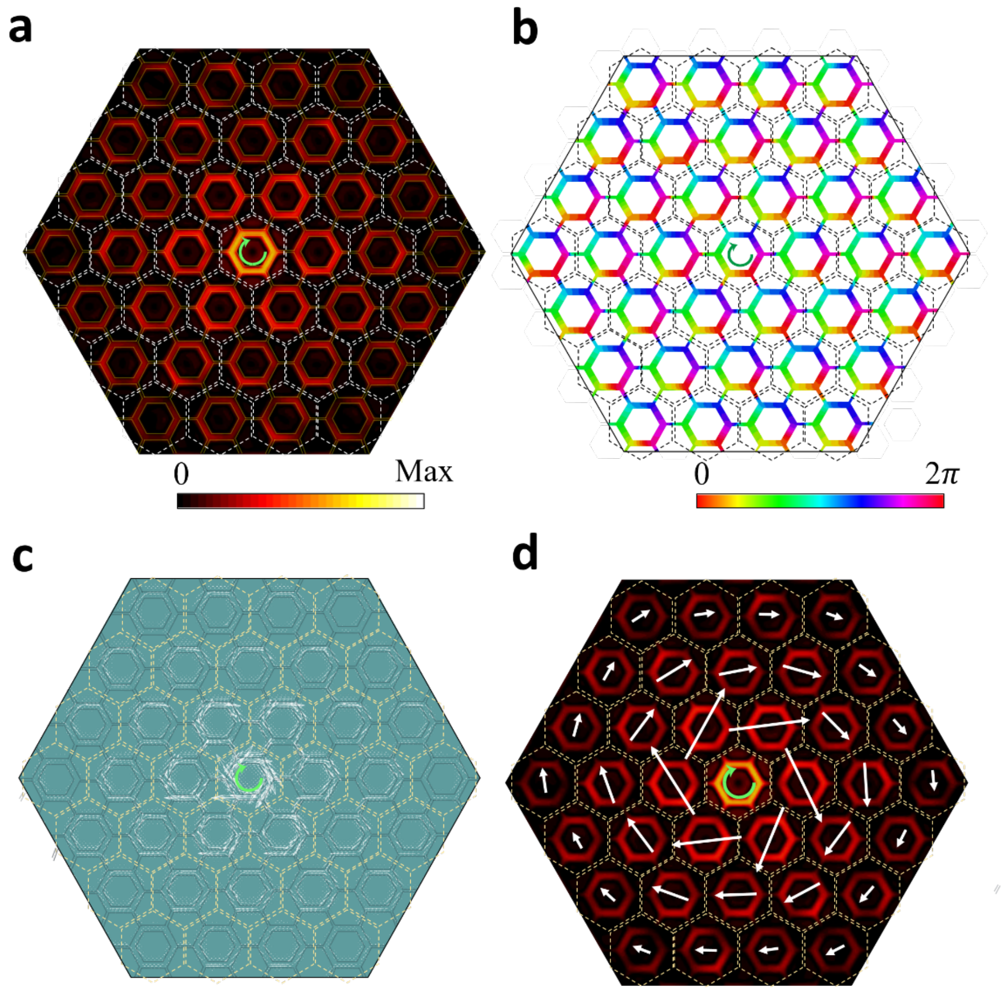

Supplementary Fig. 16. Same as Supplementary Fig. 14 but for the -2π-phase-winding chiral source. The local Poynting vectors in individual C_{6v}-symmetric unit cells rotate clockwise, which is the same as the chiral source. The Poynting vectors summed in individual C_{6v}-symmetric unit cells rotate clockwise with respect to the sample center, same to the source, corresponding to the paramagnetic chiral response.

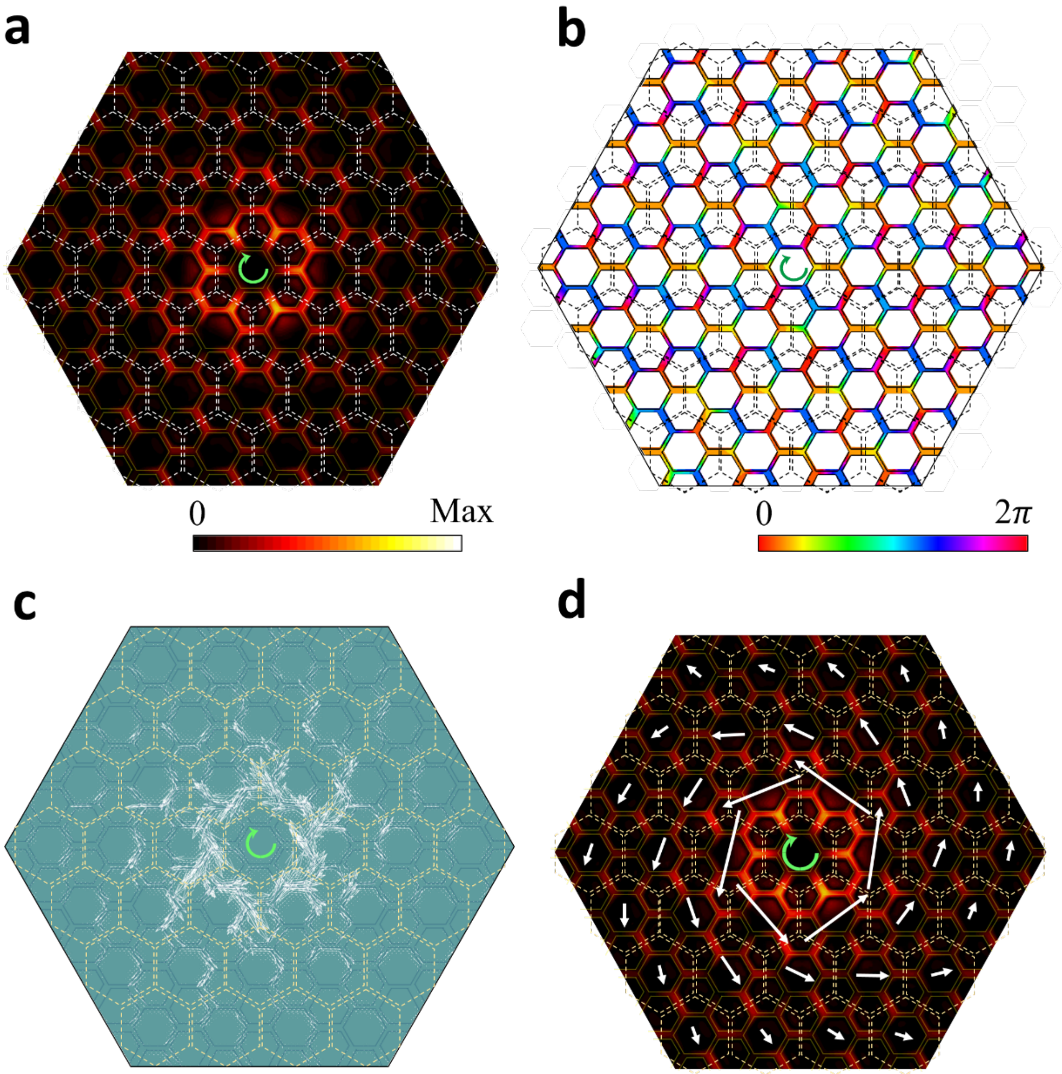

Supplementary Fig. 17. Same as Supplementary Fig. 15 but the −4π-phase-winding chiral source. The local Poynting vectors in individual C_{6v}-symmetric unit cells rotate clockwise, which is the same as the chiral source. The Poynting vectors summed in individual C_{6v}-symmetric unit cells rotate counterclockwise with respect to the sample center, opposite to the source, corresponding to the diamagnetic chiral response.

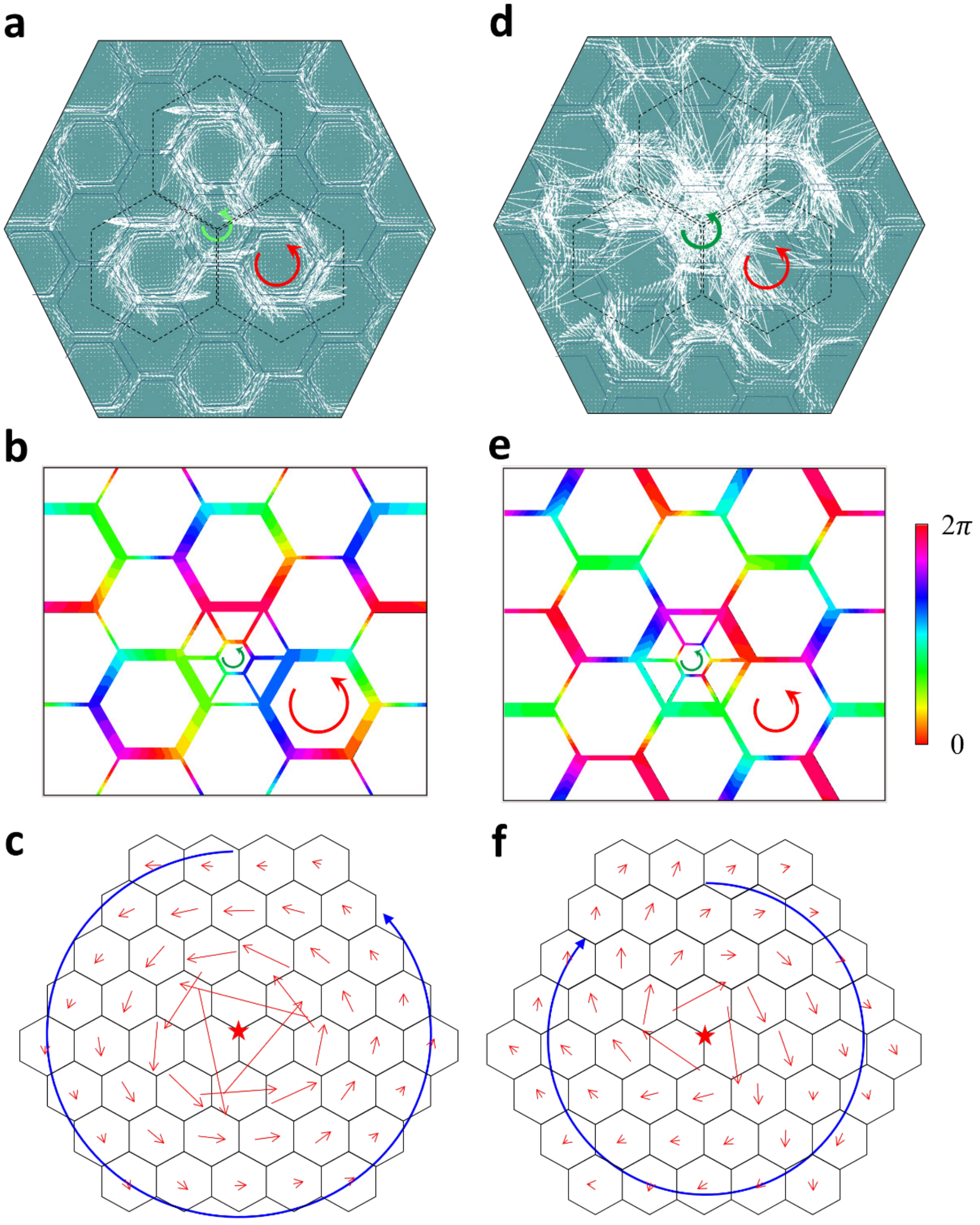

Supplementary Fig. 18. Full-wave simulation for the chiral response of topologically distinct structures when a counterclockwise phase winding chiral source is located in C_{3v}-symmetric area with frequency f = 1.42 GHz (slightly below the lower band edge). (**a**) Distribution of local Poynting vectors of the $M > 0$ structure under a +2π-phase-winding chiral source. The system with 108 C_{6v}-symmetric unit cells is exposing to air without cladding. (**b**) Distribution of the phase of

the out-of-plane electric-field E_z under the same condition as (**a**). The small hexagon at the center of the structure is the chiral source. (c) Distribution of Poynting vectors summed in individual C_{6v}-symmetric unit cells. (**d**)-(**f**) Same as (**a**)-(**c**) but for the $M < 0$ structure under the excitation of +4π-phase-winding chiral source. The chiral responses are basically same to those when the chiral source is located in the C_{6v}-symmetric unit cell.

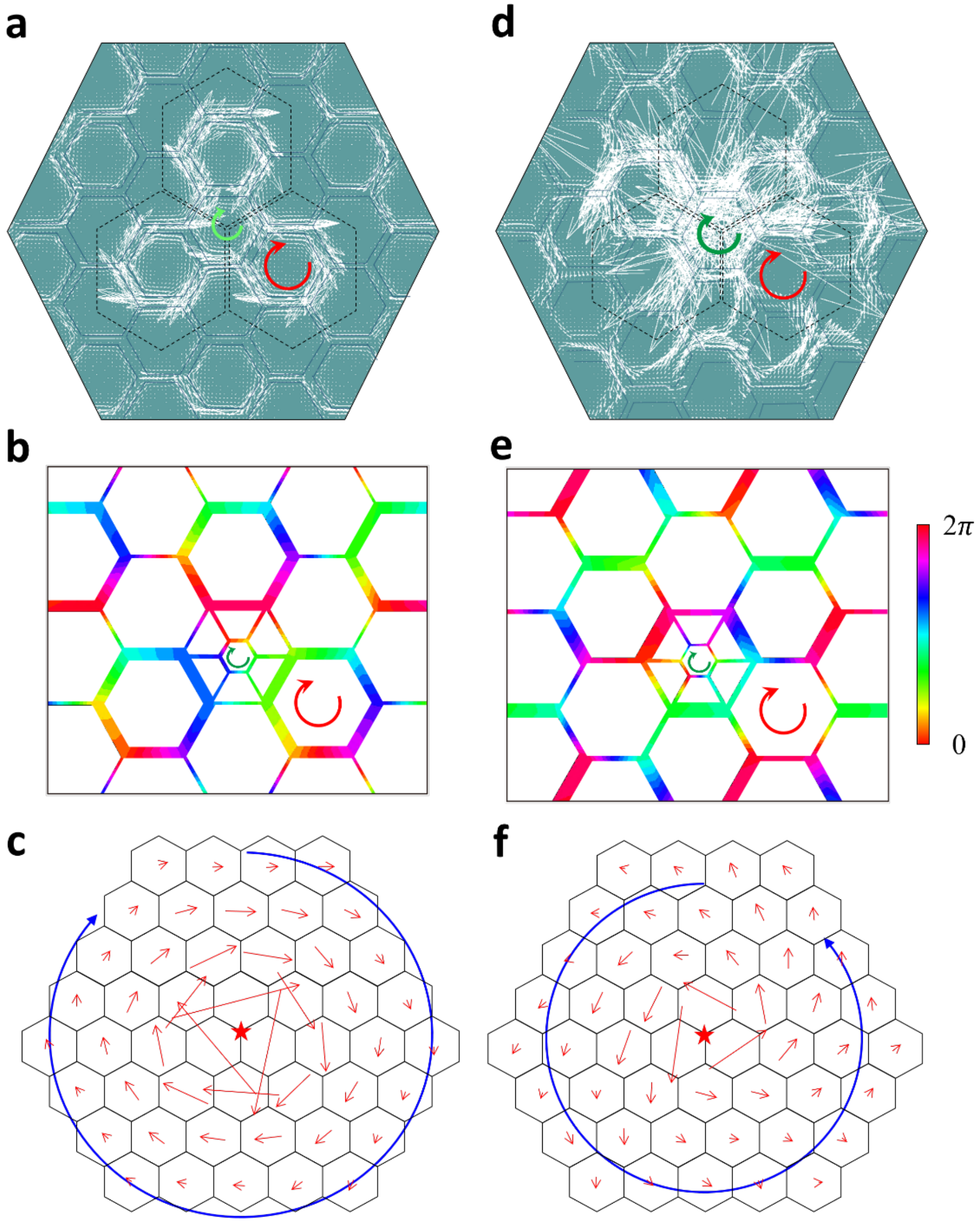

Supplementary Fig. 19. Same as Supplementary Fig. 18 but for the clockwise phase winding chiral source. The chiral responses are basically same to those when the chiral source is located in the C_{6v}-symmetric unit cell.

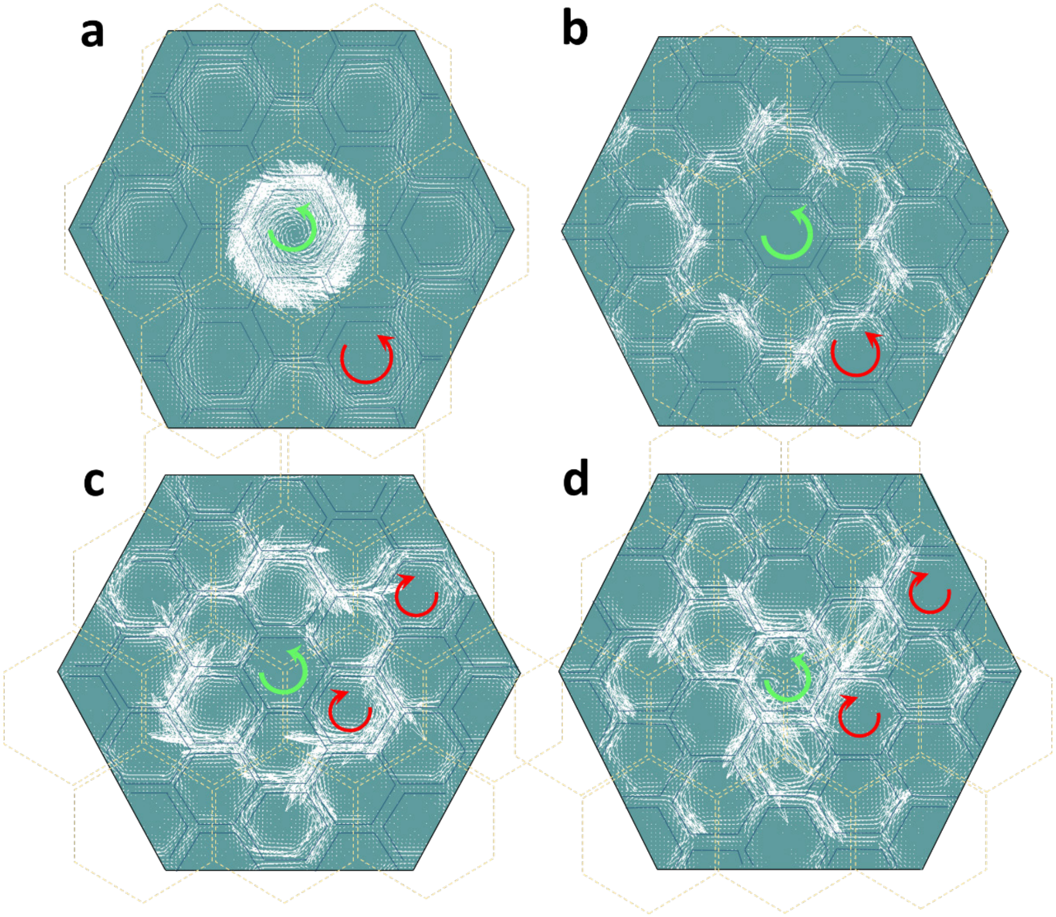

Supplementary Fig. 20. Pseudospin states induced by a counterclockwise phase winding chiral source with frequency in the band gap (f = 1.5 GHz). (**a**) Distribution of local Poynting vectors of the $M > 0$ structure under a +2π-phase-winding chiral source located in the C_{6v}-symmeric unit cell. (**b**) Distribution of local Poynting vectors of the $M < 0$ structure under a +4π-phase-winding chiral source located at the C_{6v}-symmeric unit cell. In both (**a**) and (**b**), the pseudospin is parallel to the chiral source. (**c**) (**d**) Similar to (**a**) (**b**) but for the chiral source located in the C_{3v}-symmeric area, respectively. In both (**c**) and (**d**), the pseudospin is opposite to the chiral source.

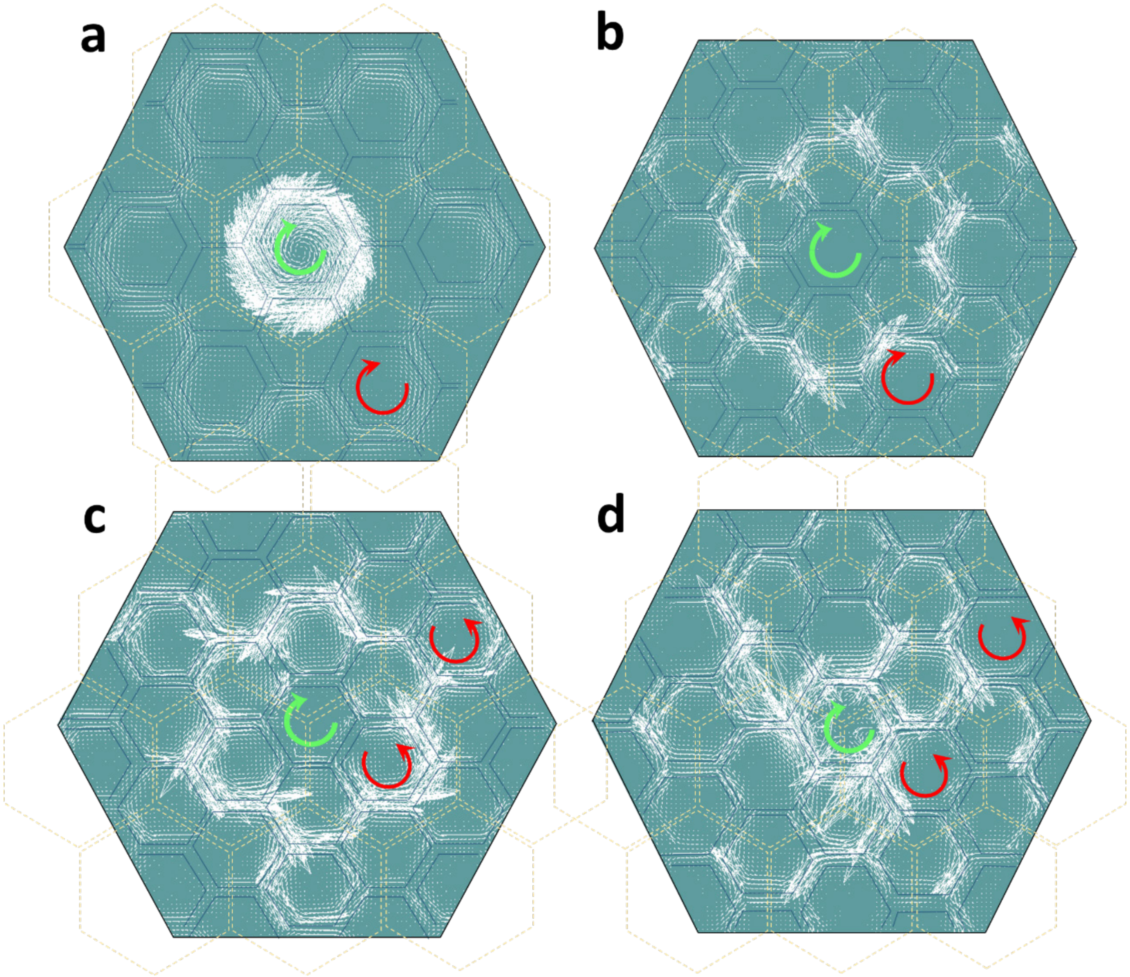

Supplementary Fig. 21. Same as Supplementary Fig. 20 but for the clockwise phase winding chiral source. The pseudospin states induced by the chiral source located in the C_{6v}-symmeric unit cell (C_{3v}-symmeric area) are parallel (opposite) to the source.

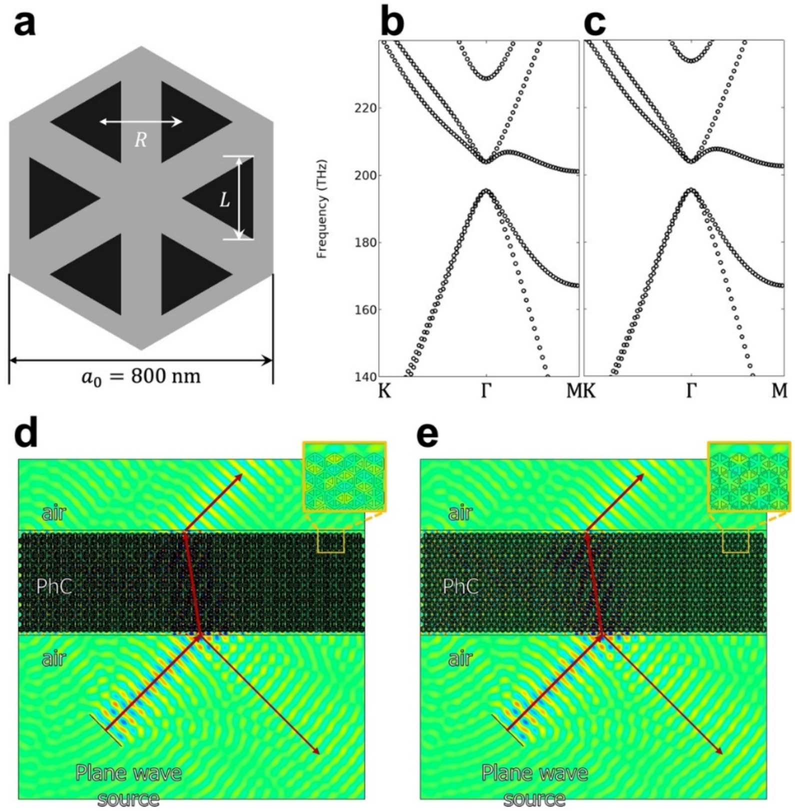

Supplementary Fig. 22. (**a**) Unit cell of the Kekulé-distorted honeycomb photonic crystal respecting $C_{6\text{v}}$-symmetry used for simulations of negative refraction. The lattice constant is $a_0 = 800$ nm. For the trivial photonic crystal, the side length of the triangle airholes is $L = 248.5$ nm and the distance between neighboring airholes in the same unit cell is $R = 246.5$ nm. For the topological photonic crystal, one has $L = 250$ nm and $R = 280$ nm. For convenience, a system with infinite thickness is adopted which reduces the simulation to two dimensions. The relative permittivity for the dielectrics is $\varepsilon = 6.6$. (**b**) and (**c**) Frequency band structure for the trivial and topological photonic crystal respectively. The lower band edges are at 190.6 THz. (**d**) and (**e**) Simulated wave propagation for an incident plane wave at 170 THz passing through a ribbon of 20 unit cells in width for the trivial and topological photonic crystal respectively. Both cases show clearly negative refraction.

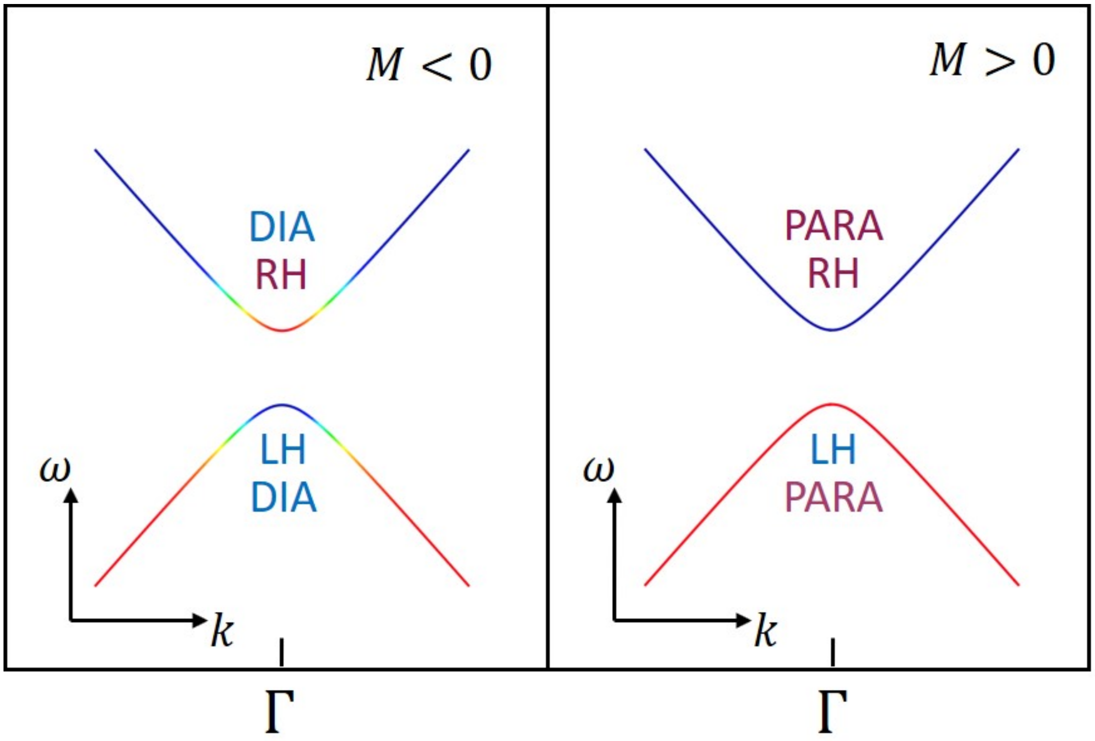

Supplementary Fig. 23. Unconventional optic properties of the Kekulé-distorted honeycomb photonic crystal, with DIA/PARA for diamagnetic/paramagnetic chiral response to chiral source and LH/RH for left-/right-handedness and negative/positive refractive index.

Supplementary References

1. Li, Y., Sun, Y., Zhu, W., Guo, Z., Jiang, J., Kariyado, T., Chen, H. & Hu, X. Topological LC-circuits based on microstrips and observation of electromagnetic modes with orbital angular momentum. *Nat. Commun*. **9**, 4598 (2018).
2. Palmer, S. J. & Giannini, V. Berry bands and pseudo-spin of topological photonic phases. *Phys. Rev. Res*. **3**, L022013 (2021).
3. Wu, L.-H. & Hu, X. Scheme for Achieving a topological photonic crystal by using dielectric material. *Phys. Rev. Lett*. **114**, 223901 (2015).
4. Long, Y., Ren, J., Guo, Z., Jiang, H., Wang, Y., Sun, Y. & Chen, H. Designing all-electric subwavelength metasources for near-field photonic routings. *Phys. Rev. Lett.* **125**, 157401 (2020).
5. Wu, L.-H. & Hu, X. Topological properties of electrons in honeycomb lattice with detuned hopping energy. *Sci. Rep.* **6**, 24347 (2016).
6. Xiao, D., Chang, M. C. & Niu, Q. Berry phase effects on electronic properties. *Rev. Mod. Phys.* **82**, 1959 (2010).
7. Veselago, V. G. The electrodynamics of substances with simultaneously negative values of ε and μ. *Sov. Phys. Usp.* **10**, 509-514 (1968).
8. Notomi, M. Theory of light propagation in strongly modulated photonic crystals: Refractionlike behavior in the vicinity of the photonic band gap. *Phys. Rev. B* **62**, 10696 (2000).
9. Cubukcu, E., Aydin, K., Ozbay, E., Foteinopoulou, S. & Soukoulis, C. M. Negative refraction by photonic crystals. *Nature* **423**, 604-605 (2003).

10. Barik, S., Karasahin, A., Flower, C., Cai, T., Miyake, H., DeGottardi, W., Hafezi, M. & Waks, E. A topological quantum optics interface. *Science* **359**, 666 (2018).

11. COMSOL Multiphysics® v. 5.6. www.comsol.com. COMSOL AB, Stockholm, Sweden.
